# SARS-CoV-2 receptor is co-expressed with elements of the kinin–kallikrein, renin–angiotensin and coagulation systems in alveolar cells

**DOI:** 10.1038/s41598-020-76488-2

**Published:** 2020-11-11

**Authors:** Davi Sidarta-Oliveira, Carlos Poblete Jara, Adriano J. Ferruzzi, Munir S. Skaf, William H. Velander, Eliana P. Araujo, Licio A. Velloso

**Affiliations:** 1 Laboratory of Cell Signaling, Obesity and Comorbidities Research Center, Instituto de Biologia - Bloco Z. Campus Universitário Zeferino Vaz, University of Campinas, Rua Carl Von Lineaus s/n, Barão Geraldo, Campinas, SP 13083-864 Brazil; 2 Physician-Scientist Graduate Program, School of Medical Sciences, University of Campinas, Campinas, Brazil; 3 Nursing School, University of Campinas, Campinas, Brazil; 4 Institute of Chemistry and Center for Computing in Engineering and Sciences, University of Campinas, Campinas, Brazil; 5 Department of Chemical and Biomolecular Engineering, University of Nebraska, Lincoln, USA

**Keywords:** Computational biology and bioinformatics, Immunology

## Abstract

SARS-CoV-2, the pathogenic agent of COVID-19, employs angiotensin converting enzyme-2 (ACE2) as its cell entry receptor. Clinical data reveal that in severe COVID-19, SARS-CoV-2 infects the lung, leading to a frequently lethal triad of respiratory insufficiency, acute cardiovascular failure, and coagulopathy. Physiologically, ACE2 plays a role in the regulation of three systems that could potentially be involved in the pathogenesis of severe COVID-19: the kinin–kallikrein system, resulting in acute lung inflammatory edema; the renin–angiotensin system, promoting cardiovascular instability; and the coagulation system, leading to thromboembolism. Here we assembled a healthy human lung cell atlas meta-analysis with ~ 130,000 public single-cell transcriptomes and show that key elements of the bradykinin, angiotensin and coagulation systems are co-expressed with *ACE2* in alveolar cells and associated with their differentiation dynamics, which could explain how changes in ACE2 promoted by SARS-CoV-2 cell entry result in the development of the three most severe clinical components of COVID-19.

## Introduction

**Figure 1:**
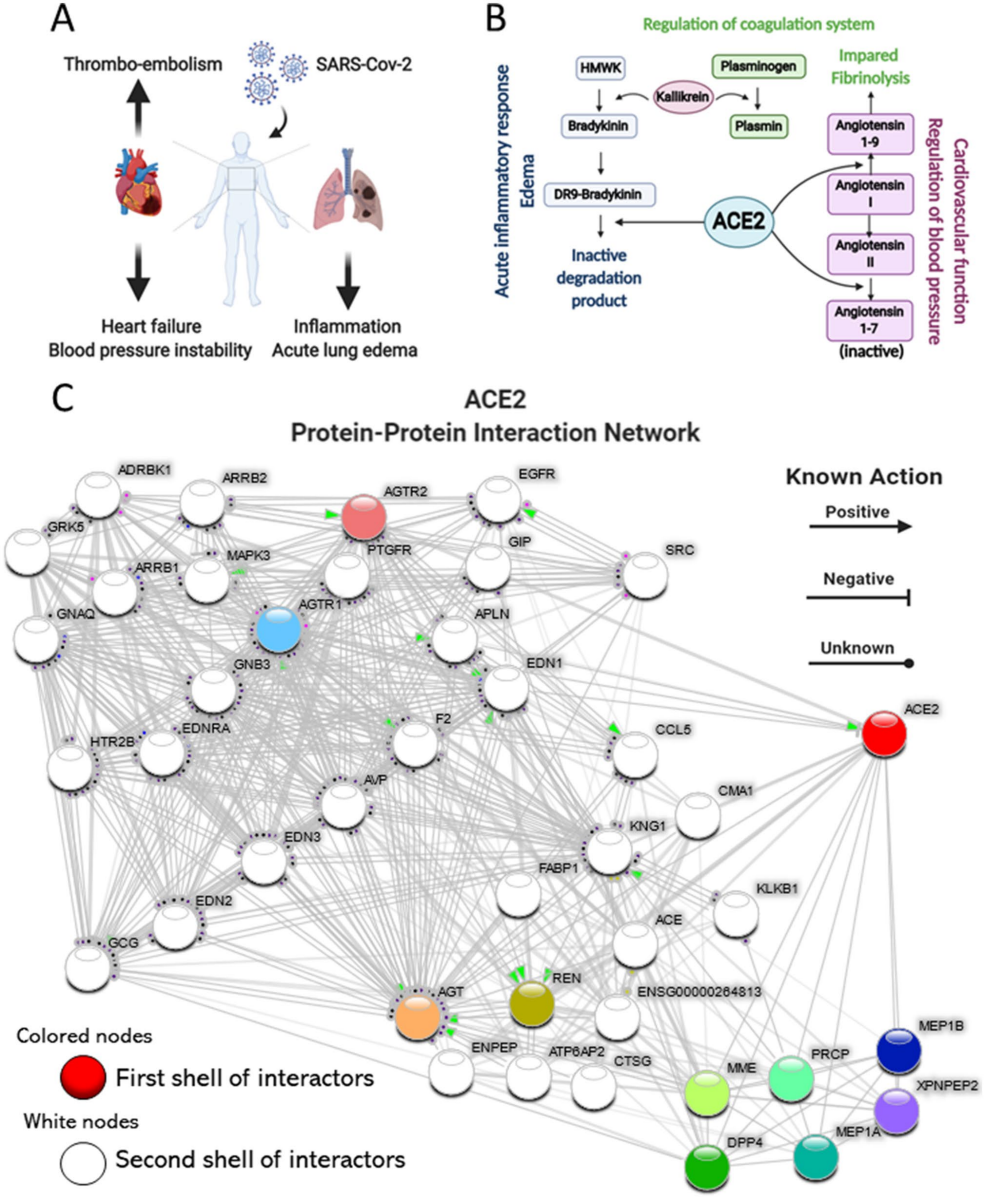
COVID-19 clinical triad and ACE2 physiological roles link COVID-19 to ACE2 dysfunction. (**A**) Schematic representation of the clinical triad that play central role in severe COVID-19. (**B**) Schematic representation of the key components of the kinin–kallikrein, renin–angiotensin and coagulation systems and their interfaces with ACE2. (**C**) ACE2 Protein–Protein Interaction Network. ACE2 interactome was retrieved with the data-mining toolkit string-db (https://version-11-0.string-db.org/cgi/network.pl?taskId=KwL0Kho7pBZf). First shell interactors of ACE2 were set as colored nodes, while second shell interactors were set as white nodes. Edges indicate known molecular action of a protein node regarding another protein node. ACE2 is directly connected to components of the kinin–kallikrein (bradykinin), renin–angiotensin and coagulation systems.

COVID-19, the disease caused by SARS-CoV-2 infection, frequently opens with cough, fever, fatigue, and myalgia^1^, progressing to a severe illness in up to 20% of infected patients^2^. Severe COVID-19 is characterized by progressive dyspnea, which results from acute lung inflammatory edema leading to hypoxia^3^. In patients surviving the initial lung inflammatory burst, a number of other complications can sum to promote a rapid and frequently lethal deterioration of health^3–5^. Acute cardiovascular failure and coagulopathy are among the most frequent complications and could be placed alongside with acute respiratory failure^3–7^ as components of a triad that leads to the highest death rates in COVID-19 (Fig. 1A).

Angiotensin converting enzyme 2 (ACE2) is the receptor for SARS-CoV-2^8^. Binding occurs through viral spike protein^9^ and depends on the serine protease TMPRSS2 for priming^8^. Physiologically, ACE2 is involved in the control of three independent but highly integrated systems, the kinin–kallikrein (KKS), renin–angiotensin (RAS), and coagulation (CS) systems (Fig. 1B). Bradykinin is a potent inflammatory substance produced from high-molecular weight kininogen (HMWK) in a reaction catalyzed by the serine protease kallikrein^10^. Bradykinin can directly deliver its vasoactive and inflammatory actions through bradykinin receptor 2 or be further processed by carboxypeptidase N to form DR9-bradykinin that activates bradykinin receptor 1 to deliver inflammatory and pain signals^11^. ACE2 degrades DR9-bradykinin into inactive peptides and, together with angiotensin converting enzyme (ACE), which inactivates bradykinin, shuts down the KKS^12^. Angiotensin II (Ang II) is a pleotropic hormone involved in the regulation of blood pressure, blood volume, cardiac function, and electrolyte balance^13,14^. It is produced from angiotensin I (Ang I) through the catalytic action of ACE, whereas ACE2 catalyzes its degradation into the inactive peptide angiotensin 1–7, thereby inactivating the RAS^15^. The interaction of ACE2 with the CS is indirect and occurs via two mechanisms: (1) catalyzing the production of angiotensin 1–9, which reduces plasminogen activator and increases PAI-1, thus inhibiting fibrinolysis^16^ and (2) modulating the activity of kallikrein, which in turn catalyzes the conversion of plasminogen into plasmin^17^.

Upon SARS-CoV-2 binding, ACE2 is internalized to endosomes, leading to a subcellular location shift that could alter its capacity to physiologically regulate the KKS, RAS and CS^18–22^. This could simultaneously impact the highly lethal COVID-19 triad: lung inflammation, cardiovascular failure, and coagulopathy. Despite the fact that ACE2 is expressed in several organs and tissues, both clinical and experimental evidence shows that SARS-CoV-2 promotes most of its pathological actions by initially infecting cells of the upper respiratory tract and, subsequently, alveolar cells in the lung^23–25^. It is currently unknown if lung cells expressing ACE2 are equipped with proteins that belong to the KKS, RAS and CS, which could potentially explain a cell-autonomous system that is disturbed by SARS-CoV-2 infection, leading to abnormal regulation of all three systems. Here, we evaluated the transcripts of 129,079 human lung cells previously submitted to single-cell RNA sequencing. We show that transcripts encoding for key elements of all three systems are highly co-expressed with ACE2 in alveolar cells.

## Results

### ACE2 interacts with proteins belonging to the kinin–kallikrein, renin–angiotensin, and coagulation systems

A protein–protein interaction network (Fig. 1C) revealed that *ACE2* interacts closely with proteins that belong to the KKS; kininogen (*KNG1*), the substrate for bradykinin synthesis, and kallikrein (*KLKB1*), the enzyme that catalyzes this conversion. *ACE2* also interacts with proteins of the RAS; *ACE*, that converts Ang I into Ang II, renin (*REN*), the enzyme that converts angiotensinogen (*AGT*) into Ang I, AGT itself and angiotensin receptor 1 (*AGTR1*). The interface of *ACE2* with CS occurs mostly though KLKB1 that controls fibrinolysis; in addition, Factor II (*F2*, thrombin), was identified in the interactome.

### Integrated analysis of 129,079 human single lung cells leads to the identification of cell types expressing *ACE2*

**Figure 2:**
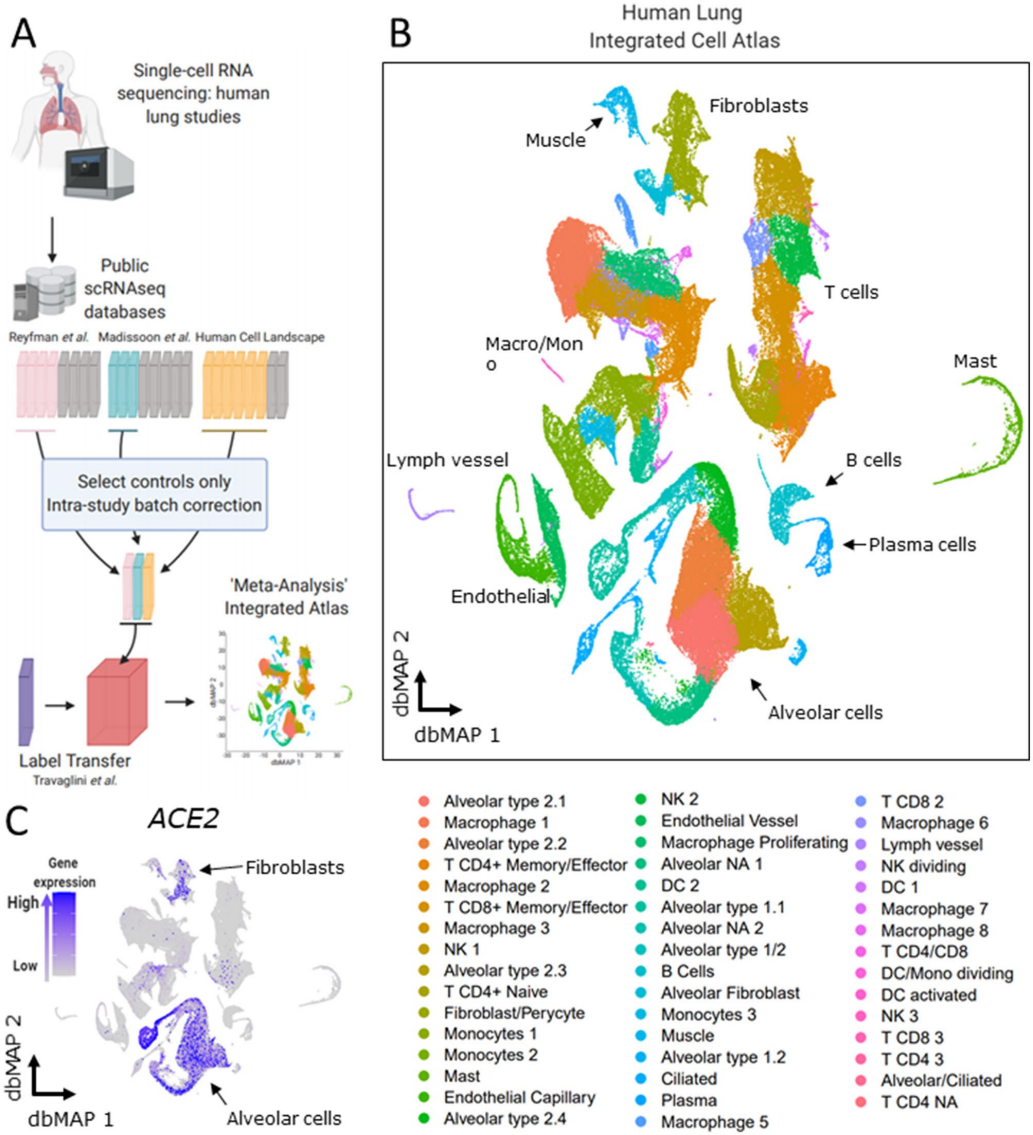
Generation of an Integrated Lung Cell Atlas through integration of public data reveal fibroblasts and alveolar cells expressing ACE2 in the human lung. (**a**) Public data from single-cell RNA sequencing (scRNAseq) studies on the human lung was retrieved. Reyfman et al., Madissoon et al. and Human Cell Landscape control datasets were individually analyzed and batch-corrected with Seurat v3 anchor-based integration after filtering and normalization. Batch-corrected data representing each study was used to assemble an integrated dataset. This integrated dataset was annotated with the assistance of label transferred from Travaglini et al. data. (**b**) The Human Lung Integrated Cell Atlas represented in two-dimensional diffusion-based Manifold Approximation Embedding (dbMAP). dbMAP organizes the visualization to preserve as much of the original data structure as possible in a comprehensive way. In this representation, each cell is a point mapped to an embedding so that its ***x, y ***coordinates represent its relative transcriptional identity, i.e. its phenotypic signal. Cells are colored by their assigned cluster. (**c**) Visualization of *ACE2* expression on the human lung dbMAP embedding. *ACE2* is consistently expressed in alveolar cells and scattered in fibroblasts, being practically absent from other cell types. *NK* Natural Killer cell; *DC* Dendritic Cell; *NA* Not Assigned.

To investigate the lung cell types that are potentially targeted by SARS-Cov-2 due to *ACE2* expression, we leveraged public single-cell RNA sequencing (scRNAseq) from three previously published datasets and a pre-print report^26–28^ (https://doi.org/10.1101/742320). Despite active investigation of the cellular landscape of the human lung, the field lacks an integrated atlas in which a consensus can be established between various datasets. We addressed this issue by individually filtering and integrating each study control sample into a batch-corrected study-wise reference with Seurat v3 anchor-based integration (Fig. 2A, Suppl. Figure 1, Suppl. Figure 2A,B, Suppl. Table 1). These batch-corrected data were used for integration of peer-reviewed studies. Preprint data was used for cell-type annotation by anchor transfer learning (https://doi.org/10.1101/742320). Corrected data were dimensionality reduced with diffusion-based Manifold Approximation and Projection (dbMAP) (https://dx.doi.org/10.2139/ssrn.3582067), a dimensionality reduction method tailored for the analysis of large-scale single-cell data and clustered by its diffusion graph structure (Fig. 2B). In this embedding, each point represents a single-cell, and the position in the embedding represents its relative transcriptional identity when compared with other cells. Grouping cells by study of origin shows that this approach is successful in removing experimental noise from data (Suppl. Figure 2B). Cells were clustered by applying the Louvain algorithm to the graph of each cell diffusion structure scoring. After removal of singletons and doublets, 47 cell clusters were identified, comprising eight major groups: alveolar cells, endothelial and lymphatic cells, monocytes/macrophages, T cells, B cells, fibroblasts, smooth muscle cells, and mast cells (Fig. 2B). We show that library size is stable across main cell types, thus discarding library-size effects from the clustering approach (Suppl. Figure 3). Clusters were then annotated based on learned annotations published elsewhere (https://doi.org/10.1101/742320) (Suppl. Figure 2C) and on cluster marker gene expression (Suppl. Figure 4). The clusters effectively correspond to biologically comprehensive cellular states that can be explored as a resource to COVID-19 studies. This potential is leveraged by dbMAP for the identification of rare cell types and cellular-state transitions in comparison against UMAP (Suppl. Figure 2D). An illustrative example is shown by B-cell differentiation into plasma cells. In the dbMAP embedding, B and plasma cells form discrete populations connected directly by intermediate cells (Fig. 2B), whereas UMAP represents these clusters as completely separated populations (Suppl. Figure 2D). We briefly explored cluster marker genes (Suppl. Figures 4, 5 and 6), albeit the exploration of this atlas exceeds the scope of this manuscript. An interactive browser with our data is available at https://humanlung.iqm.unicamp.br, allowing non-bioinformaticians to produce publication-level plots and tables in a community effort to further explore the human lung cellular landscape. We next investigated the expression of *ACE2* and show that it is restricted to alveolar cells and fibroblasts, being practically absent from other cell clusters (Fig. 2C).

**Figure 3:**
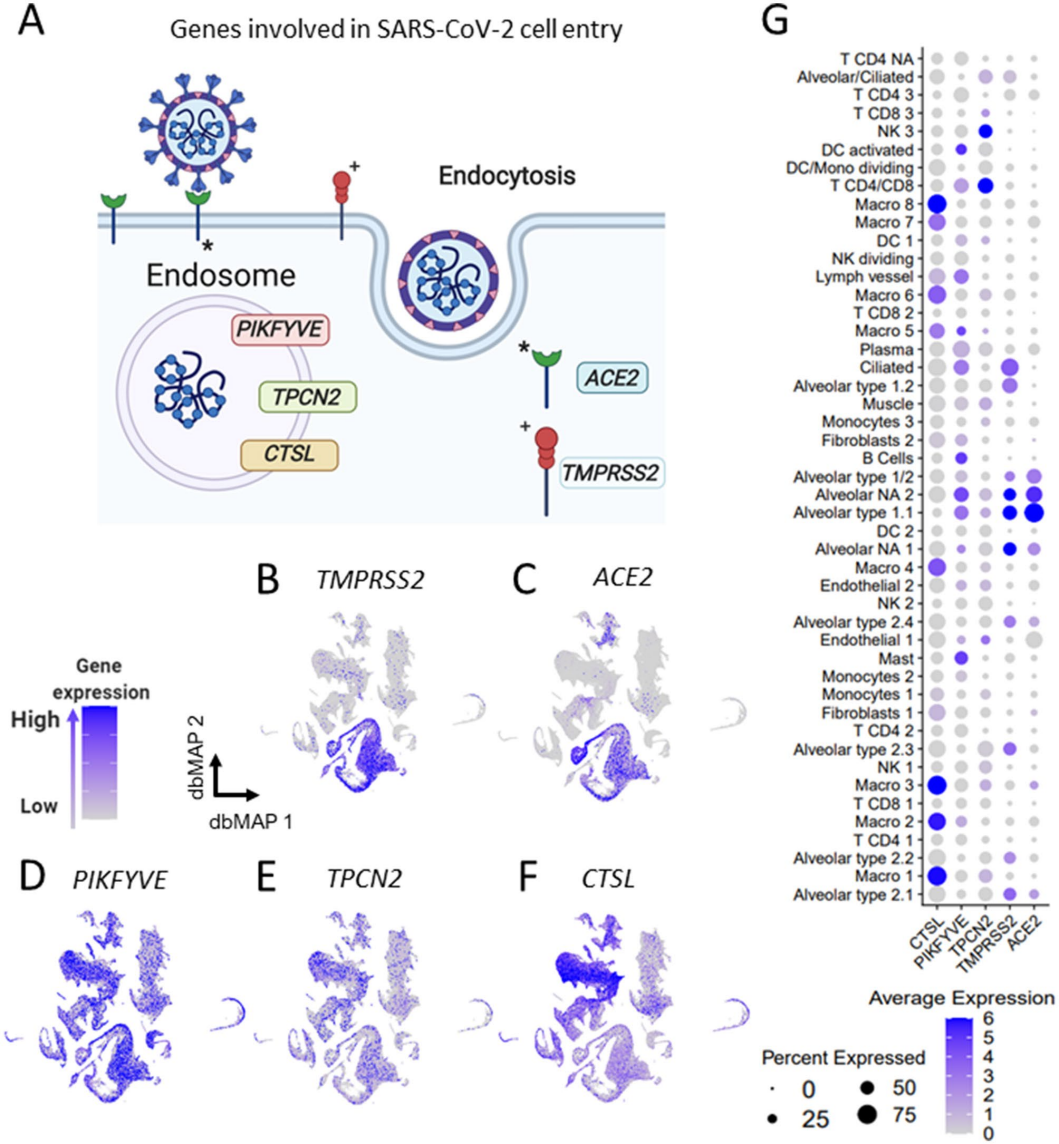
Lung expression of genes involved in SARS-Cov-2 cell entry is selective to alveolar cells. (**a**) Schematic representing SARS-Cov-2 cell entry mechanism. TMPRSS2 cleaves SARS-Cov-2 *spike* protein, allowing it to bind to ACE2, its functional receptor. It is known that other genes such as *PIKFYVE*, *TPCN2* and *CTSL* also play a role in SARS-Cov-2 endocytosis and cellular contamination (**b-f**). Visualization of gene expression in dbMAP embeddings of the lung atlas. **b.** *TMPRSS2* expression is restricted to alveolar cells. **c.** *ACE2* expression is selective to alveolar cells and fibroblasts. (**d**–**f**) *PIKFYVE*, *TPCN2* and *CTSL* are expressed consistently throughout the majority of lung cell types. *CTSL* expression in macrophages (**g**). Dot plot visualization of expression of genes involved in SARS-Cov-2 cell entry in human lung cell types. *ACE2* expression is selective to alveolar clusters, similarly to *TMPRSS2*, but with the particularity of being lowly expressed in a large portion of endothelial cells. *PIKFYVE*, *CTSL* and *TPCN2* are expressed by all clusters, but *CTSL* expression is much higher in macrophages, while *TPCN2* expression is higher in NK and T CD4/CD8 clusters.

**Figure 4:**
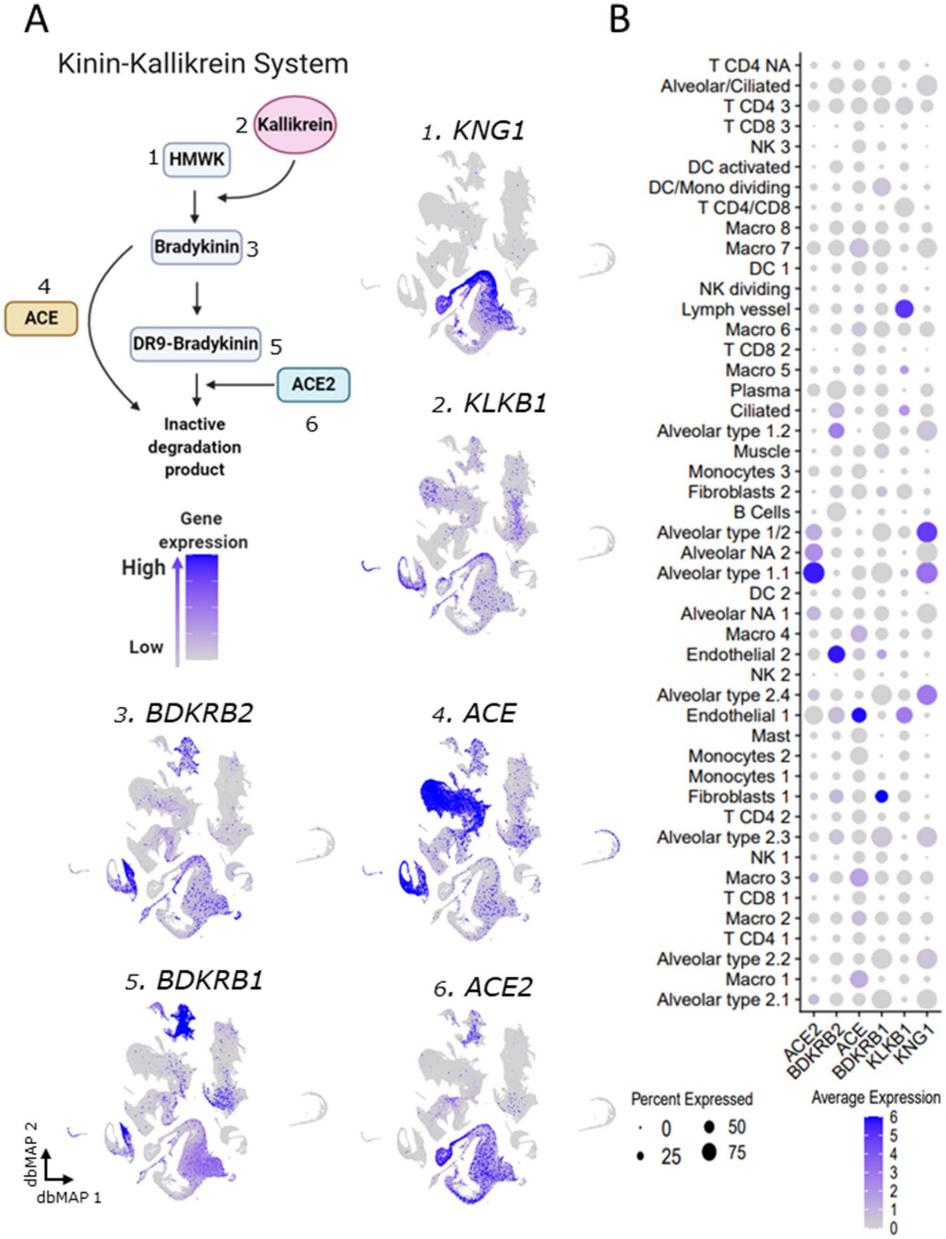
Gene expression of components of the kinin–kallikrein system point to alveolar cells role. (**a**) Schematic representing the main players and steps of the bradykinin system, also known as the kinin–kallikrein system. High molecular weight kininogen (*KNG1*) conversion into bradykinin is catalyzed by kallikrein. Bradykinin binds to the bradykinin receptor 2 (*BDKRB2*), but it can be converted into DR9-bradykinin, which activates the bradykinin receptor 1 (*BDKRB1*) until it is degraded by ACE2 into inactive degradation products. Bradykinin can also be directly converted into inactive degradation products by ACE. **1**–**6** Visualization of gene expression in dbMAP embeddings of the lung atlas. **1** *KNG1* is selectively expressed in alveolar cells. **2** *KLKB1* is expressed in endothelial and lymph vessel cells, and sparsely expressed in alveolar cells. **3** *BDKRB2* expression is selective to endothelial cells. **4** *ACE* expression is selective to endothelial cells and macrophages. **5** *BDKRB1* expression is selective to endothelial cells and fibroblasts, but also detected in low levels in alveolar cells. **6** *ACE2* expression is restricted to alveolar cells. (**b**) Dot plot visualization of expression of genes involved in the bradykinin system in the lung atlas cell types. *ACE2* and *KNG1* gene expression is selective to alveolar clusters, despite *ACE2* being lowly expressed in a large portion of endothelial cells and *KNG1* also being lowly expressed in a large portion of clusters of macrophages. *KLKB1* is highly expressed in endothelial and lymph vessel cells, but also lowly expressed in T CD4 clusters. *BDKRB2* is highly expressed in endothelial and alveolar type 1.1 cells, and also less expressed by a large portion of B/plasma cells, and of macrophages clusters 7 and 8. *ACE* is selectively expressed in endothelial cells and in macrophages. *BDKRB1* expression is selective to fibroblasts, although various clusters express it in low levels. *ACE2* expression is restricted to alveolar cells.

**Figure 5:**
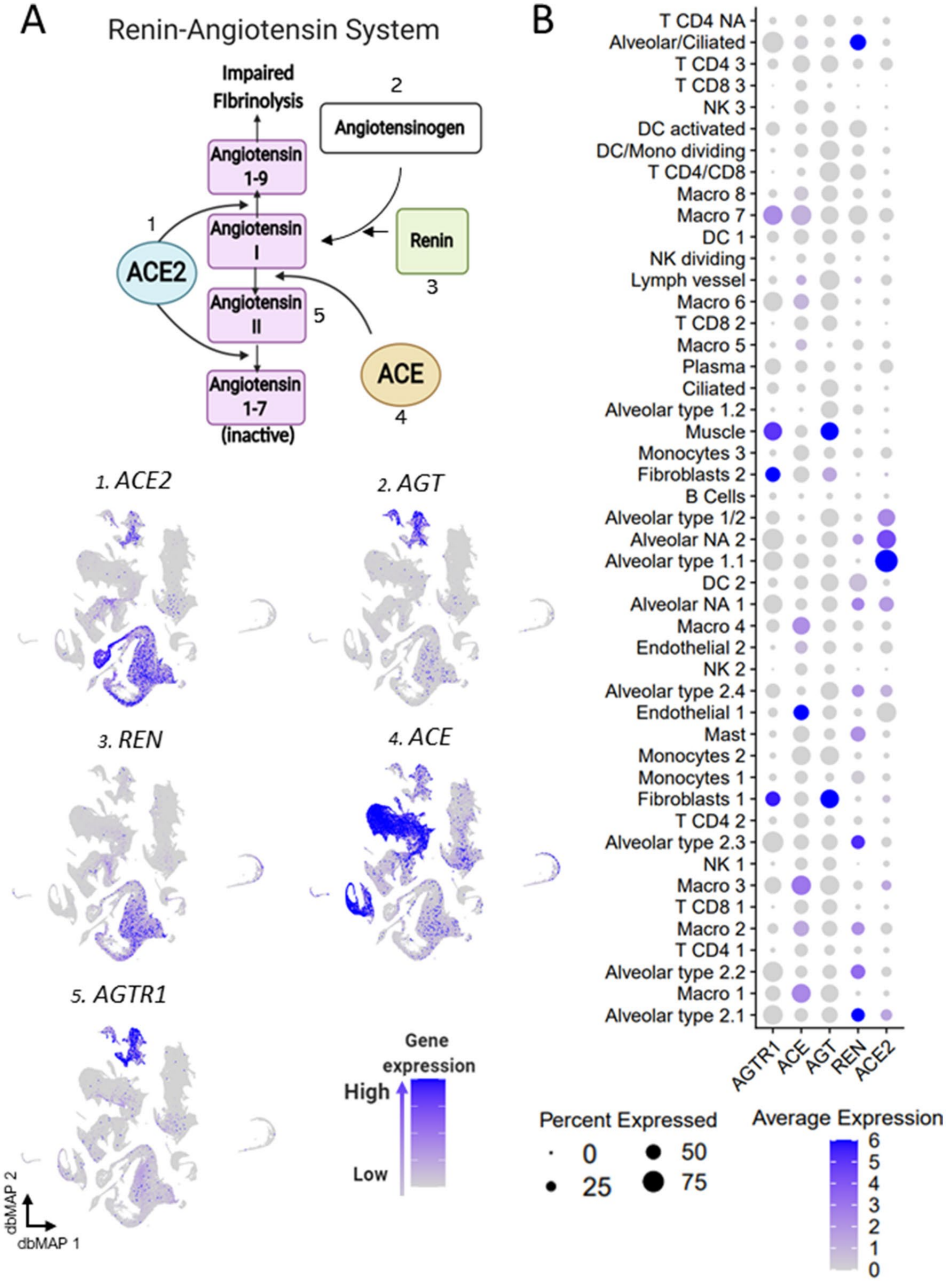
Gene expression of components of the renin–angiotensin system point towards alveolar cells role. (**a**) Schematic representing the main players and steps of the angiotensin system, also known as the renin–angiotensin system (RAS). ACE2 catalyzes the conversion of angiotensin I to angiotensin 1–9, and of angiotensin II into inactive angiotensin 1–7. Renin catalyzes the conversion of angiotensinogen (*AGT*) into angiotensin I. ACE catalyzes the conversion of angiotensin I into angiotensin II, which binds to its receptor AGTR1. **1**–**5** Visualization of gene expression in dbMAP embeddings of the lung atlas. **1** *ACE2* expression is restricted to alveolar cells. **2** *AGT* is expressed in fibroblasts and smooth muscle cells. **3** *REN* expression is restricted to alveolar cells. **4** *ACE* expression is selective to endothelial cells and macrophages. **5** *AGTR1* expression is selective to fibroblasts and smooth muscle cells. (**b**) Dot plot visualization of the expression of genes involved in the angiotensin system in the lung atlas cell types. *ACE2* expression is restricted to alveolar cell clusters, similarly to *REN*, which is virtually absent from remaining clusters. *AGT* is highly expressed in fibroblasts and muscle, albeit being lowly expressed in a large portion of cells from other clusters. *ACE* is preferentially expressed in endothelial cells and macrophage clusters. *AGTR1* is highly expressed in smooth muscle cells and fibroblasts, and lowly expressed in macrophages and alveolar cells.

**Figure 6:**
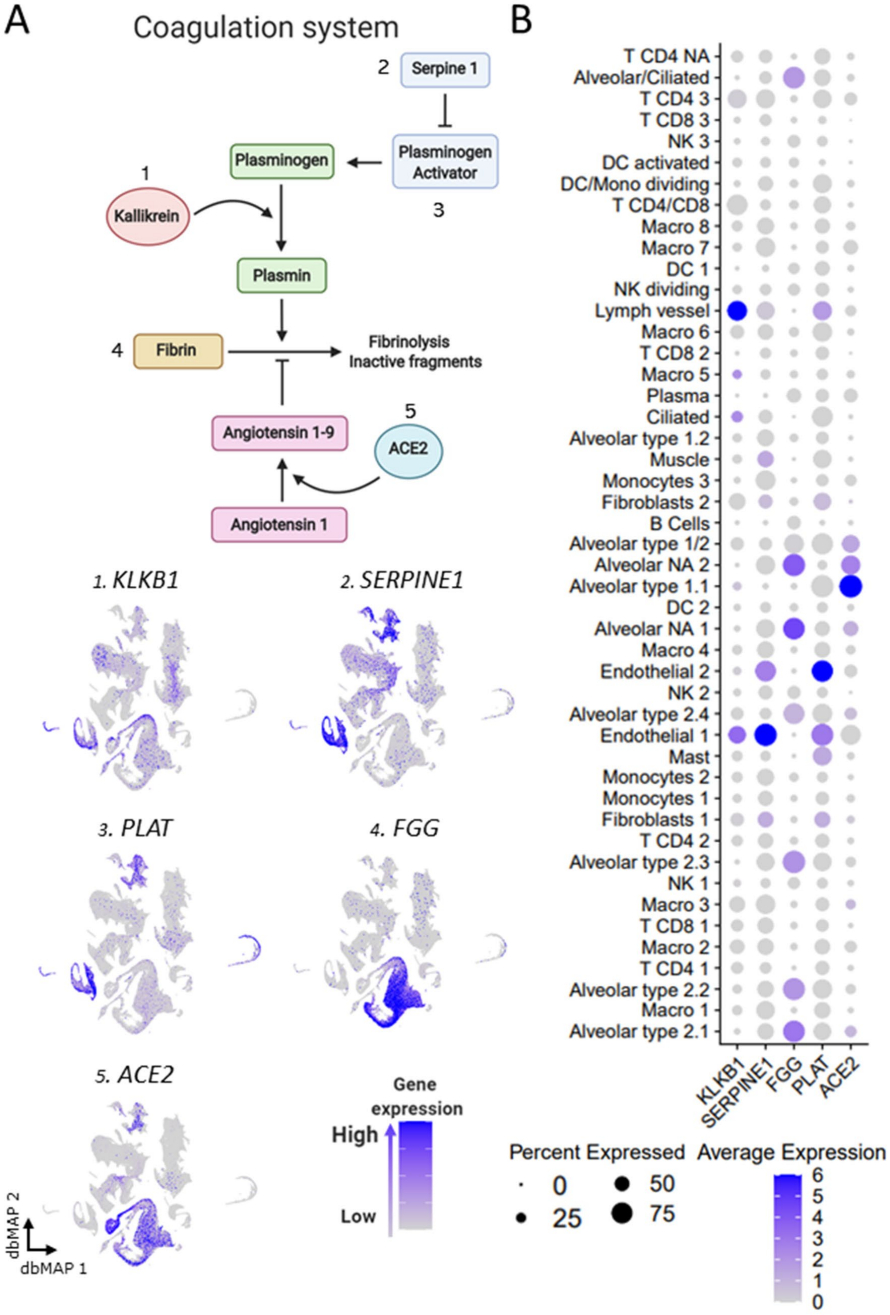
Gene expression of components of the coagulation system. (**a**) Schematic representing the final step of the coagulation system. Plasma kallikrein (*KLKB1*) catalyzes the conversion of plasminogen into plasmin. Plasminogen is activated by tissue plasminogen activator (*PLAT*), which is regulated by serpine 1 (*SERPINE1*). Plasminogen conversion into plasmin promote fibrinolysis by degradation of fibrin networks generated from fibrinogen (*FGG*) into inactive fragments. ACE2 plays a role in this process by catalyzing the conversion of angiotensin I to angiotensin 1–9, which inhibits fibrinolysis. **1**–**5** Visualization of gene expression in dbMAP embeddings of the lung atlas. **1** *KLKB1* is expressed in endothelial cells and sparsely in alveolar cells. **2** *SERPINE1* is highly expressed in fibroblasts and in endothelial and smooth muscle cells, and present in lower levels in macrophages. **3** *PLAT* is mostly expressed in endothelial cells and fibroblasts. **4** *FGG* expression is restricted to alveolar cells. **5** *ACE2* expression is restricted to alveolar cell clusters. (**b)** Dot plot visualization of the expression of genes involved in the coagulation system in the lung atlas cell types. *ACE2* expression is restricted to alveolar cell clusters. *PLAT* expression is higher in endothelial cells, lymph vessel cells and mast cells. *FGG* expression is restricted to alveolar cell clusters, being higher in alveolar type 2.1 and type 2.2, alveolar/ciliated and alveolar NA clusters.

### *TMPRSS2* is co-expressed with *ACE2* in alveolar cells

The serine protease TMPRSS2 is required for SARS-CoV-2 spike protein priming and subsequent binding to ACE2^8^ (Fig. 3A). Following receptor binding, SARS-CoV-2 is internalized through endocytosis in a process that depends on the activity of phosphatidylinositol 3-phosphate 5-kinase (*PIKFYVE*) and its downstream effector, two-pore channel subtype 2 (*TPCN2*)^18^ (Fig. 3A). Moreover, the inhibition of cathepsin L (*CTSL*) dramatically reduces virus entry, suggesting a role for this lysosome protein in the process^18^ (Fig. 3A). Out of the four proteins currently described as players in SARS-CoV-2 cell entry, only *TMPRSS2* gene expression selectively overlapped with *ACE2* expression (Fig. 3B,C,G). *TMPRSS2* transcripts are expressed in virtually all alveolar cell clusters in addition to ciliated and alveolar/ciliated clusters. As expected for a phosphatidylinositol 3-phosphate kinase, *PIKFYVE* is ubiquitously expressed throughout all lung-cell clusters, with some predominance in clusters of alveolar type 2 cells, B-lymphocytes, and mast cells (Fig. 3D,G). Likewise, *TPCN2* is ubiquitously expressed in lung-cell clusters, albeit in fewer cells than *PIKFYVE* and some predominance in T-lymphocytes and NK cells (Fig. 3E,G). *CTSL* is also expressed in virtually all lung-cell clusters. However, here there is a large predominance in macrophage clusters (Fig. 3F,G).

### Kininogen and kallikrein expressions are restricted to alveolar cells

Kininogen (*KNG1*), the precursor for bradykinin synthesis, is selectively expressed in alveolar cells, which collectively also collectively express ACE2 (Fig. 4A1,A6,B). *KNG1* expression is restricted to alveolar type 1.1, type 1/2, and type 2.4, in addition to alveolar 2.2. and 2.3 clusters. Kallikrein (*KLKB1*) is a serine protease that catalyzes the conversion of kininogen into bradykinin. It is expressed predominantly in endothelial and lymph vessels cells (Fig. 4A2,B). *KLKB1* is also expressed in scattered alveolar cells and in some T-lymphocytes and macrophages (Fig. 4A2). Bradykinin acts through the type 2 cognate receptor (*BDRK2*), which is expressed predominantly in endothelial vessel cells and in some alveolar cells, particularly types 1.2 and 2.3 (Fig. 4A3,B). *BDRK2* is also expressed in a considerable number of fibroblasts and plasma/B-lymphocytes (Fig. 4A3,B). Bradykinin can either be converted into inactive kinins by the catalytic action of ACE or be converted into the active DR9-bradykinin. *ACE* is expressed predominantly in macrophages, monocytes, and endothelial cells (Fig. 4A4,B). It is also expressed in scattered alveolar cells, T-lymphocytes, and fibroblasts (Fig. 4A4,B). DR9-bradykinin acts through bradykinin receptor 1 (*BDRK1*), which is predominantly expressed in fibroblasts, endothelial cells, and alveolar type 2.1, 2.2, and 2.3 cells (Fig. 4A5,B).

### Renin and angiotensin receptor 1 are co-expressed with ACE2 in alveolar cells

Angiotensinogen (*AGT*) is the precursor for Ang I; we show that most cells expressing *AGT* are fibroblasts and smooth muscle cells; however, a considerable number of type 2.4, 2.3, and 2.1 alveolar cells that express *ACE2* also express *AGT* (Fig. 5A1,A2,B). Renin is the enzyme that converts *AGT* into Ang I; here, we show that in the lung it is predominantly expressed in alveolar cells and largely co-expressed with ACE2, particularly in alveolar cell types 1.2, 2.2, 2.3, 2.4, and NA 2 (Fig. 5A1,A3,B). Some macrophages and mast cells also express renin (Fig. 5A3,B), which is virtually absent in the remaining clusters. Ang I is converted into active Ang II by ACE (Fig. 5A4,B), which is largely expressed in macrophages and endothelial cells. Ang II exerts most of its cardiovascular effects by acting through angiotensin II receptor 1 (*AGTR1*). We show that most cells expressing *AGTR1* are fibroblasts and muscle cells (Fig. 5A5,B); in addition, a considerable number of alveolar type 1.1 and NA 2 cells, which express *ACE2,* also express *AGTR1* (Fig. 5A1,A5,B).

### Fibrinogen gamma is co-expressed with *ACE2* in alveolar cells

In addition to its action on the KKS, kallikrein (*KLKB1*) acts in the CS by converting plasminogen into the fibrinolytic substance plasmin; *KLKB1* is expressed predominantly in endothelial and lymph vessel cells and in alveolar type 1.1, type 1/2, and type 2.4 cells (Fig. 6A1,B). Another catalyst of plasmin production is tissue plasminogen activator (*PLAT*), which is inhibited by PAI-1 (*SERPINE1*). *SERPINE1* is expressed predominantly by endothelial cells and secondarily by fibroblasts and muscle cells (Fig. 6A2,B), whereas *PLAT* is expressed in endothelial and lymph vessel cells (Fig. 6A3,B). Fibrinogen gamma chain (*FGG*) is the substrate for production of fibrin, the main component of clot formation; it is expressed in virtually all alveolar cell types except for types 1.1 and 1.2, thus largely coinciding with the expression of *ACE2* (Fig. 6A4,A5,B).

### Molecularly resolving the human alveolar epithelial cell lineage reveals that ACE2 is associated with the differentiation of alveolar type 1 cells

**Figure 7:**
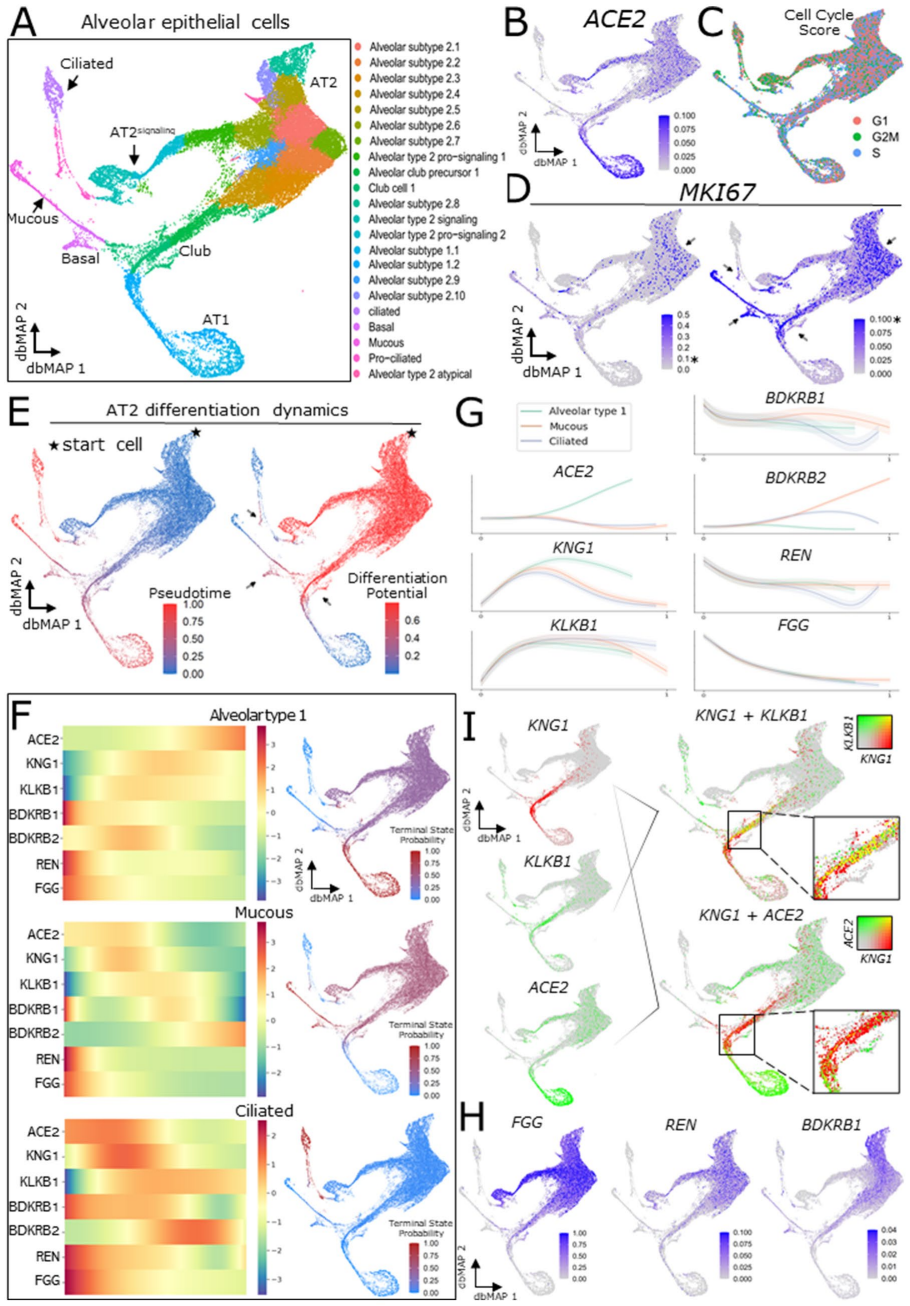
ACE2 and components of the KKS, RAS and CS are differentially expressed throughout alveolar epithelium differentiation dynamics. (**a**) Two-dimensional dbMAP embedding of 37,449 alveolar cells transcriptomes. Cells are colored by their assigned subtype. Main cell types are indicated. (**b**) Visualization of *ACE2* expression in the dbMAP embedding. The expression pattern prioritizes AT1 cells, and less so AT2 cells. (**c**) Cell cycle scoring of each single-cell and its assigned cell-phase. Note how all phases are detected across the embedding, albeit at different proportions. (**d**) *MKI67* expression visualization in the dbMAP embedding with two different coloring thresholds. AT2 cells express *MKI67* at higher levels (left; arrow), and mucous, basal, club and pro-ciliated cells present lower, yet consistent levels of *MKI67* expression (right; arrows). The * indicates a fixed expression level, for easier visualization. (**e**) AT2 progeny differentiation dynamics. The star represents the start cell used in computations. Left: pseudotime score for each cell, i.e. how much each cell is advanced in the differentiation trajectory. Right: differentiation potential for each cell, i.e. the probability that cell *i* transit into an adjacent cell *j,* also seen as *entropy.* Note that the intermediate states (pointed by arrows) present a higher differentiation potential than terminally differentiated cells. (**f**) The three terminal states detected for the AT2 progeny—alveolar type 1 (AT1), mucous and ciliated cells. The heatmaps on the left column indicate relative expression changes (left to right) throughout the differentiation trajectories shown on the right column that correspond to terminal states. (**g**) Gene expression trends of *ACE2* and components of the KKS, RAS and CS expressed in alveolar cells. *ACE2* expression is directly associated with the maturation of AT1 cells, whereas *KNG1* and *KLKB1* are preferentially expressed in intermediate states. *REN*, *FGG* and *BDKRB1* are preferentially expressed in AT2 progenitors at the beginning of the differentiation process, and *BDKRB2* expression is directly associated with the differentiation mucous cells. (**i**) Visualization of co-expression (yellow) by coloring overlap. *KNG1* (red) is co-expressed with *KLKB1 (*green, top) in intermediate states corresponding to club cells, but barely co-expressed with *ACE2* (green, bottom). (**h**) Visualization of *FGG, REN and BDKRB1* expression in the dbMAP embedding. As shown in (**g**), these genes are preferentially expressed by AT2 progenitors, being undetected in terminally differentiated states.

Investigation of stem cell activity in adult lung homeostasis and regeneration is a rapidly expanding field with solid evidence for cellular regeneration in adult humans^29^. A number of studies have explored the stem cell function of alveolar type 2 and basal cells expressing *KRT5* in different contexts, mostly relying on in-vitro or animal models^30–33^; while others have used scRNAseq technologies to gain insights on basal cell differentiation dynamics in different in-vitro settings^34^ and on neuroendocrine cell regeneration in the adult mouse lung^35^. Collectively, these studies have generated extensive insight into the alveolar epithelium lineage; however, a systems-wise, comprehensively defined lineage is yet to be identified. Here, we addressed this issue by exploring alveolar cells from our meta-analysis reference of the healthy human adult lung and proceeded with subsetting of alveolar clusters from the integrated atlas, subsequently re-embedding it with dbMAP to further investigate their transcriptional heterogeneity (Fig. 7A). After a clustering approach similar to the one employed on the complete atlas, we identified 22 alveolar cell subtypes, corresponding to five main phenotypes: alveolar type 1, alveolar type 2, mucous, basal and ciliated cells. Next, we profiled *ACE2* expression in these lineages and discovered that it is restricted to alveolar type 1 and type 2 cells (Fig. 7B). To obtain an insight on cell cycle activity within alveolar cells, we performed gene enrichment analysis of phase marker genes to infer each cell phase in the cycle (Fig. 7C). We found that cells transit throughout cycle phases within the embedding structure, with a higher number of G2M cells in alveolar type 2 and pro-ciliated cells, and intermediate states between alveolar type 2 cells and terminally differentiated mucous and alveolar type 1 cells. Next, we explored cells selectively expressing *MKI67*, a canonical marker of the G2M/S phase, with two different color scale thresholds to define putative progenitor clusters (Fig. 7D). Using this approach, we show that *MKI67 *expression occurs predominantly in alveolar type 2 cells and, at lower levels, in alveolar type 2-signaling, club, basal and mucous cells. Next, we employed Palantir^36^ to assess pseudotemporal ordering of cells taking either alveolar type 2 (Fig. 7E) or basal cells (Suppl. Figure 7A) as the starting cluster. Remarkably, the structure encoded on the dbMAP embedding highlights cellular transitions and intermediate states between well-defined clusters, similarly as seen with B cell differentiation into plasma cells, with terminally differentiated states elegantly corresponding to the extremities of the embedding, whereas such fine information is not preserved by UMAP (Fig. 7E, Suppl. Figure 7B). As shown, alveolar type 2 cells present robust differentiation potential towards three terminally differentiated states corresponding to ciliated, alveolar type 1, and mucous cells (Fig. 7E,F). Progenitors differentiate into a common transit-amplifying state corresponding to club cells, which in turn give rise either to alveolar type 1 or mucous cells, strikingly matching recent results that show that a subpopulation of club cells mobilize to promote alveolar regeneration^37^. This branching alveolar type 1-mucous lineage is phenotypically different from the alveolar type 1-ciliated lineage, although both present *MKI67* expressing transit-amplifying progenitors and differentiated, steady-state progenitors which present high terminal state probability (Fig. 7E,F). From this perspective, *KRT5*+ basal cells can be understood as an intermediate steady-state, which preferentially give rise to mucous cells (Suppl. Figure 7A,C), similarly to ciliated and alveolar type 1 transit-amplifying progenitors (Fig. 7E,F). The dynamics underlying basal cell proliferation are poorly understood in the healthy lung, and its study is frequently associated with fibrosis and viral respiratory diseases, which significantly differs from our healthy-controls-only scenario. Based on these findings and the differences between the basal cell progeny literature and our settings, we decided to perform further analysis considering the alveolar type 2 progeny, which express *MKI67* at higher levels. We point that basal cells could be preferentially involved in mucous and alveolar type 1 cell regeneration in the context of lung injury—a potential aspect to be explored in further studies employing scRNAseq of samples from deceased COVID-19 patients. As our findings differ from previous studies which pointed AT2 cells as the main target cell type for SARS-Cov-2, we also show that AT1 cells preferentially co-express *ACE2* and *TMPRSS2*, while only a few AT2 did so (Suppl. Figure 7D).

### KNG1 and KLKB1 are selectively co-expressed in alveolar type 1 club progenitors with low ACE2 expression

**Figure 8:**
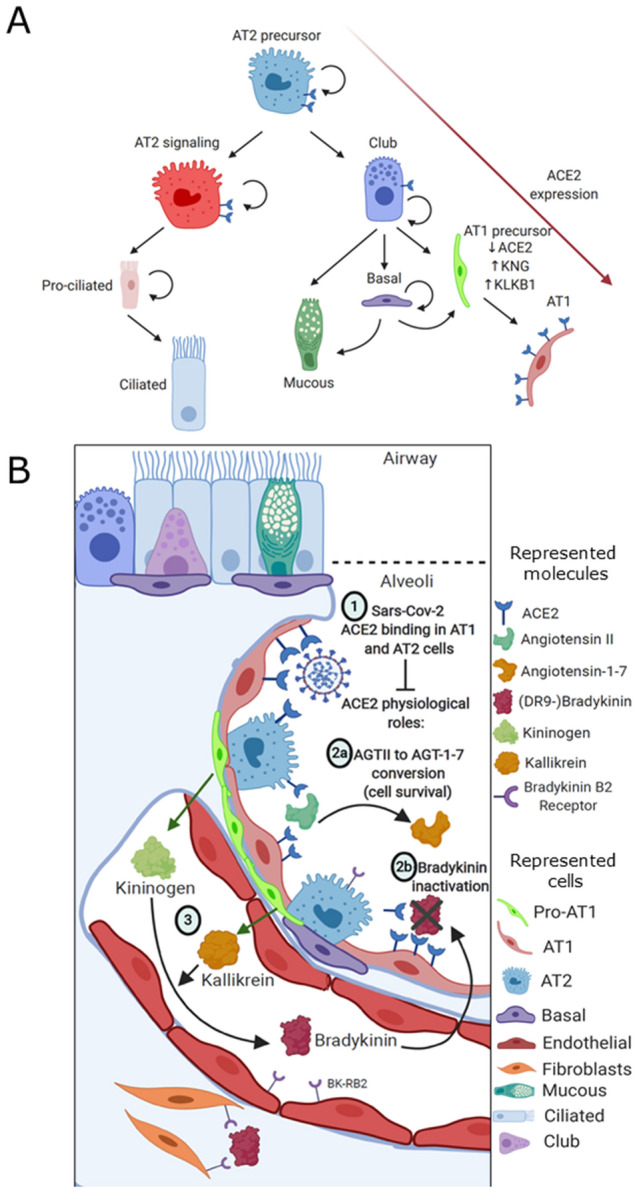
A theoretical model for Sars-Cov-2 pathogenesis. (**a**) Schematic representation of the identified alveolar epithelium progeny, with AT2 precursors giving rise to club and AT2-signaling cells. The latter then gives rise to pro-ciliated and ciliated cells, whereas the latter give rise to basal, mucous and AT1 precursor cells. AT1 precursors which are *ACE2-/KNG1*+ */KLKB1*+ give rise to mature AT1 cells, which highly express ACE2 during their differentiation process. Selective disruption of the alveolar niche turnover dynamics could thus be implicated in Sars-Cov-2 pathogenesis, and we encourage further studies investigating these associations. (**b**) Schematic representation of the healthy alveolus prior to Sars-Cov-2 binding to *ACE2* (1). Either by structural *ACE2* changes or by disruption of the *ACE2*+ population, Sars-Cov-2 infection potentially interferes with two key *ACE2* roles: (2a) converting Ang II to Ang 1–7, thus compromising alveolar cell survival signaling; and (2b) degradation of bradykinin into inactive products. Pro-AT1 (club) cells highly and selectively express kininogen and kallikrein (3), which jointly leads to the production of bradykinin. Kallikrein is also involved in converting plasminogen into plasmin (not shown). Infection derived disruption of the alveolar niche with selective preservation of *ACE2-/KNG1*+ */KLKB1*+ pro-AT1 club cells could be associated with disturbance of the KKS, leading to severe inflammation and altered coagulation.

We then explored the gene expression trends of *ACE2* and genes involved in the KKS, RAS, and CS (Fig. 7G), and found *KNG1* and *KLKB1* to be selectively co-expressed in club cells corresponding to intermediate states between alveolar type 2 precursors and differentiated alveolar type 1 and mucous cells. (Fig. 7G,I). We investigated whether cells expressing *KNG1* and *KLKB1* also expressed *ACE2* and found that this population presented consistently lower levels of *ACE2* compared to alveolar type 2 progenitors and terminally differentiated alveolar type 1 cells (Fig. 7I). We also found that alveolar type 2 progenitors selectively express *REN, FGG*, *BDKR1, ACE and AGT* (Fig. 7H, Suppl. Figure 7E)*,* while expression of *BDKRB2* was mostly restricted to mucous cells (Suppl. Figure 7F). Clonal expansion of alveolar progenitors has been previously associated with lung disease progression and fibrosis, being also proposed as an indirect measure of lung injury. It has been shown that abnormal alveolar proliferation upon injury is involved in lung disease progression and fibrosis, although the mechanisms by which this occurs are more elusive. The fact that such intermediate progenitors can cell-autonomously elicit activation of the KKS and their absence of *ACE2* expression points to SARS-Cov-2 selectively targeting steady-state and terminally differentiated alveolar cells rather than intermediate states, and selective preservation of these pro-inflammatory states could contribute to the impact of alveolar niche disruption. Moreover, these results implicate *ACE2* and the KKS in the differentiation dynamics and turnover of the alveolar epithelium, which remain largely unexplored in the context of COVID-19. Finally, we built a hypothetic framework for SARS-Cov-2 lung injury based on the interaction of the KKS, RAS and CS with the differentiation dynamics of alveolar epithelia (Fig. 8A,B), in a first of its kind mechanism that couples the illness clinical components to cell-specific phenotypes which impact in lung injury and in circulating inflammatory and coagulation factors.

## Discussion

Here, we present the first meta-analysis of the healthy human lung transcriptional landscape. This was achieved by the careful data re-analysis and assembly of previous independent studies, each with its own batch-derived bias, into an integrated cell atlas containing 129,079 cells^26–28^. We further leveraged the power of this data by analyzing it with dbMAP, a novel dimensional reduction method that, together with the integrated dataset, are publicly available at (https://github.com/davisidarta/humanlung and https://github.com/davisidarta/dbMAP). An user-friendly interactive web-based platform is also available at https://humanlung.iqm.unicamp.br, in a powerful data exploration environment that holds potential to accelerate lung research. This data can also be used by future studies performing scRNAseq and data analysis of the human lung, so that others may have the option to add their study into this integrated atlas. Using this approach, we confirmed previous data that identified alveolar cells as those expressing highest levels of the SARS-CoV-2 receptor, ACE2, in the lung^23^. In addition, we identified cell types that express transcripts encoding for proteins potentially involved in SARS-CoV-2 cell entry. We demonstrated that gene expression of *TMPRSS2*, a serine protease that primes SARS-CoV-2 spike protein, largely overlaps with *ACE2* expression in alveolar cells^23^, reinforcing the hypothesis of TMPRSS2 as a promising pharmacological target of COVID-19^38^. Moreover, we showed that transcripts encoding for PIKFYVE, TPCN2, and CTSL^18^ are ubiquitously expressed in the lung and, even though they are expressed in alveolar cells with some degree of overlap with *ACE2*, their potential as therapeutic targets could be challenged due to lack of cell-type specificity.

Next, we determined the identities of lung cells expressing key components of the KKS, RAS, and CS pathways. Currently, there is neither experimental nor clinical evidence suggesting that SARS-CoV-2 infection could lead to bradykinin-dependent lung inflammation and edema. However, in a rodent model of lung LPS-induced inflammation, the inhibition of ACE2 resulted in a significant increase lung inflammatory edema, and the simultaneous inhibition of bradykinin in this model dramatically reduced inflammatory damage^12^. In addition, in humans, severe lung edema can develop in a rare genetic disorder, hereditary angioedema, that results from bradykinin accumulation due to mutations of the C1 esterase/kallikrein inhibitor^39^. Clinical studies have shown that inhibitors of bradykinin can efficiently treat lung edema in these circumstances^40^. Furthermore, the use of ACE inhibitors provides yet another line of clinical evidence for disturbed lung accumulation of bradykinin that can, on rare occasions, lead to severe outcomes^41,42^. Here, we showed that the gene encoding HMWK (*KNG1*) is consistently co-expressed with that encoding kallikrein (*KLKB1*), which converts HMWK into bradykinin, in a particular set of alveolar type 1 progenitors. This population presents low ACE2 expression compared to AT2 progenitors and mature alveolar type 1 cells, and could be potentially preserved from viral infection, leading to an unbalance in the alveolar niche towards a pro-inflammatory phenotype.

In contrast to KKS, abnormalities in the RAS have been widely reported in COVID-19 patients^43–45^. Obesity, hypertension, diabetes, and coronary insufficiency represent the greatest risk factors for severe COVID-19^5,6^, and in all these diseases, there is an increased risk for abnormal regulation of the RAS^13,46,47^. In one of the largest series reporting COVID-19 patients published so far, the use of ACE inhibitors before infection was shown to reduce mortality by 33%, representing the most effective independent factor, among those analyzed, that could protect patients from a lethal outcome^6^. Here we showed that angiotensinogen, the precursor of Ang 1, and particularly renin, the enzyme catalyzing this conversion, are preferentially expressed in *ACE2* expressing alveolar type 2 progenitors. In addition, other key components of the RAS, as *ACE*, *AGT*, *AGT1R1* and *REN*, are also selectively expressed in this ACE2 + alveolar population.

COVID-19-associated coagulopathy can lead to a fulminant activation of coagulation, resulting in widespread thrombosis. Venous thromboembolism (VTE) is one of the leading causes of severe complications in COVID-19 patients^3,48^. It was diagnosed in 20% of patients admitted to an intensive care unit (ICU) and its cumulative incidence increased progressively to 42% as patients remained in severe condition in the ICU^48^. Developing VTE during the progression of severe COVID-19 increases the risk of death by 140%^48^. Moreover, D-dimer, a degradation product of fibrin, has been identified as an important predictive marker of severe COVID-19 and was directly correlated with poor prognosis^49,50^. Because of the association between disturbed coagulation and severe disease progression, the use of anticoagulant treatment has been proposed as potentially beneficial in COVID-19^22,51^. In one therapeutic clinical trial, the use of low-molecular-weight heparin resulted in reduced mortality in patients with high levels of D-dimer^52^; however, in another study, preventive anticoagulation was associated with increased VTE^53^. These results suggest that prophylactic and therapeutic anticoagulation approaches have distinct outcomes in COVID-19; however, further studies are awaited in order to direct the establishment of optimal measures that could lessen the severity of COVID-19 coagulation abnormalities. Here, we showed that FGG expression is particularly restricted to alveolar type 2 progenitors in a similar pattern as of ACE and REN. Both FGG and KLKB1 play central roles in the control of the CS. FGG deficiency leads to mild or even severe bleeding^54^, whereas KLKB1 deficiency is associated with an increased risk of thrombosis^55^.

We further explored the alveolar epithelium transcriptional heterogeneity and *ACE2* expression pattern and show for the first time that *ACE2* is consistently expressed in alveolar type 1 cells and directly associated with their differentiation dynamics. This finding adds to previous work that has shown *ACE2* expression in alveolar type 2 and alveolar type 1 cells^56^. We also show that alveolar type 1 precursors selectively co-express *KNG1* and *KLKB1*, which are needed for bradykinin production, while being practically void of ACE2 expression, in contrast to alveolar type 2 progenitors and terminally differentiated alveolar type 1 cells. A previous study^57^ has shown a considerable loss of the alveolar type 1 cell population in the histological analysis of deceased COVID-19 patients, which presents diffuse alveolar damage with fibrin-rich hyaline membrane formation and reactive pulmonary hyperplasia. While the totality of our analysis places loss of lung ACE2 activity at the center of a theoretical basis for COVID-19 pathogenesis, whether SARS-Cov-2 exerts these effects by changing ACE2 expression or function remains an unanswered question. Yet, as SARS-Cov-2 patients present expressive loss of alveolar type 1 cells and alveolar epithelial hyperplasia, we hypothesize that SARS-Cov-2 ultimately carries its effects in the lung through preferential targeting of the ACE2+ alveolar population, particularly ACE2^high^ alveolar type 1 cells, leading to ACE2 loss of function. In such a setting, ACE2 physiological roles would be impaired, leading to increased Ang-2 pro-apoptotic activity and reduced Ang1-7 cell survival signaling in addition to increased kallikrein activity and bradykinin signaling that could induce cell death and population imbalance in the alveolar niche. We note that the exact proportion of alveolar type 1 and alveolar type 2 cells in human alveoli and whether it changes upon SARS-Cov-2 infection is unknown; however, alveolar type 1 cells are generally considered to be more numerous than alveolar type 2 cells. We also show that ACE2+ alveolar type 1 precursors express transcripts encoding high-molecular weight kininogen and kallikrein, adding to previous observations that expanded alveolar cells can develop into pro-inflammatory phenotypes, such as in other lung affections as pulmonary fibrosis and influenza infection. It is long known that quantification of damage induced to the alveolar epithelium can be performed by measuring alveolar cell proliferation^58^. As alveolar hyperplasia was observed in severe COVID-19 histology^57^, it is reasonable to assume that SARS-Cov-2 promotes abnormalities in the KKS, RAS and CS that are least partly derived from a reactional increase in alveolar type 1 precursors which produce serum KKS elements, and a decrease in terminally differentiated ACE2^high^ alveolar type 1 cells, responsible for ultimately inhibiting the KKS through ACE2 activity. The accumulation of in situ and systemic bradykinin in SARS-Cov-2 patients is still unknown, but it could be one of the mechanisms contributing to the severe inflammatory activity in COVID-19, as suggested elsewhere^59^.

The major limitation of this work relies on the fact that we were unable to perform experiments with lung samples from COVID-19 patients. We were also limited to gene expression information, and whether that is properly accompanied by increased protein content upon SARS-Cov-2 infection is yet unclear. Further studies investigating whether alveolar turnover is in fact disrupted by SARS-Cov-2 are needed, as well as further measures of ACE2 activity and Ang II, Ang 1–7 and bradykinin blood and lung concentrations in severe illness.

In conclusion, *ACE2* expression is restricted to alveolar cells, which are primary cellular targets for SARS-CoV-2 in the lung. These cells also express transcripts encoding proteins that play pivotal roles in the regulation of the KKS, RAS, and CS. Together with *ACE2*, these transcripts are involved in the differentiation process of alveolar epithelia during adult lung cell turnover, and differentially expressed throughout progression of stem-like states to terminally differentiated epithelial cell types. As all these systems are potentially affected during the progression of severe COVID-19, we propose that disruption of the alveolar niche and abnormal function of ACE2 as a result of SARS-CoV-2 infection could directly and cell-autonomously precipitate the development of lung injury, acute inflammation, cardiovascular failure and thromboembolism, which are the hallmarks of severe COVID-19^55^.

## Methods

### Computational environment

In silico analyses were performed on two different machines. Locally, a ThinkPad P52 Workstation with 128 GB RAM and a six-core Intel Xeon processor was used for data exploration. A high-performance computing cluster (HPCC) was used for data integration and computation of results. Analysis was performed in R version 3.6.2. A docker environment with a pre-installed RStudio image and loaded with all required packages is available at https://github.com/davisidarta/lung_covid.

### Protein–protein interaction networks

Protein functional interaction networks were performed with STRING v11^60^, a software toolkit which performs datamining on a large number of databases and on individually published high-throughput datasets. The default functional interaction network was queried for ACE2 in the *Homo sapiens* organism and was visualized by known molecular action. We subsequently added a second shell of interactors in order to explore deeper interactions through autonomous datamining. A permalink webpage of ACE2 Protein–Protein Interaction network is accessible through https://version-11-0.string-db.org/cgi/network.pl?networkId=KwL0Kho7pBZf .

### Single-cell RNA sequencing data acquisition, filtering, and processing

Control samples (healthy donors) digital expression matrices were downloaded from Gene Expression Omnibus for data published elsewhere^26^ (GSE122960) and for Human Cell Landscape (HCL) data^27^ (GSE132355). For other datasets^28^, raw digital expression matrices and associated metadata were downloaded from the Human Cell Atlas web portal (https://data.humancellatlas.org/explore/projects/c4077b3c-5c98-4d26-a614-246d12c2e5d7). In addition, preprint data from Travaglini and coworkers (https://doi.org/10.1101/742320) was obtained from synapse (Human Lung Cell Atlas—https://www.synapse.org/#!Synapse:syn21041850/wiki/600865). Cells were filtered by total number of reads *(nreads),* by number of detected genes (*ngenes*), and by mitochondrial percentage (*mito.pc*). Data filtering thresholds were defined by inflection points in the logarithmic curves of each cell’s number of reads (*nreads*) and number of detected genes (*ngenes*). Filtering was performed on each individual sample prior to integration, with the exception of the study by Madissoon et al.^28^, in which each sample corresponds to a different point in a time course of frozen storage of cells prior to processing (0–72 h), rendering extremely low batch effects. Filtering was performed in each individual sample prior to integration, with the exception of Madissoon et al. study^28^, in which each sample corresponds to a different point in a time-course of frozen storage of cells prior to processing (0–72 h), rendering extremely low batch effects. Supplementary Table 1 summarizes the thresholds used by each sample in each individual study, and Supplementary Fig. 1 summarizes quality-control metrics for each dataset.

Each individual sample was processed in Seurat v3.1.5^61^ using the default Seurat workflow. For each individual sample, cell counts were log-normalized by a size factor of 10,000 RNA counts, and feature selection was performed by selecting the 5,000 genes with the highest dispersion. Data were then scaled (z-core transformation to standardize expression values for each gene across all cells). For sample batch-correction within each study, we performed the combination of the gene expression of each sample and then selected 2,000 features to be used during the integration process prioritizing features that were identified as highly variable in multiple samples. Unsupervised identification of anchor correspondences between the Canonical Correlation Analysis (CCA) space of each sample normalized data was performed with the *FindIntegrationAnchors* function in Seurat v3 with default parameters. These anchors were scored and weighted, being subsequently merged into integrated assays containing the balanced expression of the union of each sample’s expressed genes, resulting in three study-wise balanced datasets.

### Data integration and label transfer

For integration of data from different studies, we performed the union of the gene expression of available human lung peer-reviewed studies^26–28^. Prior to anchoring, 3,000 features were selected by ranking top features identified as highly variable in multiple studies. We proceeded similarly to the individual study level, where anchors were built between multiple samples, and searched for anchor correspondences between each study’s own manifold by using Seurat v3 Integration. As a first step, dimensional reduction with CCA is performed. CCA effectively captures gene modules that are correlated and shared in pair-wise dataset comparisons, representing signals that define a shared biological state. Afterwards, mutual nearest neighbors (MNN—pairs of cells, each from one dataset) that share a corresponding state are weighted in an ‘anchor’, which is scored so as to exclude spurious connections between unrelated cell types. The integration of these anchors into a single manifold was performed with the *IntegrateData* function, and the top 20,000 genes with higher dispersion were included in the final result. The data of Travaglini et al. (https://doi.org/10.1101/742320) were not included in the integrated atlas due to its lack of peer review. However, we did use these data annotations for label transfer due to its high-quality cell-type annotation. This was performed by employing the FindTransferAnchor and TransferData functions, in which a classification matrix is transposed by multiplication by a weighting matrix to return prediction scores for each class for every cell in the atlas. Those labels were further used as guidance in the process of cell-type annotation, as well as the expression of cell-type marker genes.

### Visualization of library-size effects

For the visualization of library-size effects, we proceeded with an alternative clustering strategy in which the resolution parameter was lowered to 0.3 (default 0.8), rendering main cell types as clusters. We then computed the sum of detected genes in the integrated data and show that it is constant across different main cell types. Similar results were obtained when analyzing all presented subclusters (data not shown). We also note that we obtained a higher number of detected genes than those detected in each study, pointing towards single-cell data integration reported ability to impute sparse signals from individual datasets.

### Dimensionality reduction with diffusion-based manifold approximation and Projection (dbMAP)

Visualizing single-cell data is a challenging task in which comprehensive two- or three-dimensional embedding needs to be generated from an exceptionally large number of samples and observations. Previous approaches mainly relied on performing prior dimensionality reduction with PCA to denoise data and make it computationally easier to compute non-linear dimensional reduction methods such as t-stochastic neighborhood embedding (t-SNE), UMAP, and potential of heat diffusion for affinity-based trajectory embedding (PHATE). We have recently proposed diffusion-based Manifold Approximation and Projection (dbMAP), which excels at identifying rare cell populations and describing lineage dynamics as trajectories progress, branch, and cycle (https://dx.doi.org/10.2139/ssrn.3582067). Briefly, dbMAP encodes and denoises data by dissecting its diffusion structure, in which cell–cell similarity information is adaptive regarding each cell’s individual neighborhood density. This information is then propagated through a series of random walks, which are scaled to normalize the relative progression of diffusion during each walk. This approach effectively adapts diffusion maps to approximate the Laplace–Beltrami operator, which represents data intrinsic structure. The resulting eigenvectors are diffusion components which can be used for downstream clustering, layout visualization and pseudotime estimation, being a potential substitution for PCA in single-cell processing. These diffusion components address UMAP’s assumptions on its input, thus optimizing its potential for visualization, a feature we explored with dbMAP. dbMAP is computationally scalable and robust to variations in parameters, uniquely allows the visualization of cyclic trajectories in which stem cells actively traverse the cell cycle and is well-suited for the analysis of organ- and organism-level data. dbMAP takes four main parameters. During diffusion, a number **N** of structure components are computed accounting for each cell **K** nearest neighbors. After automatic scaling and selection of relevant components by eigengap analysis, a UMAP layout is generated with **M** as the effective minimum distance between embedded points and **S** as the effective scale of embedded points. Importantly, visualization parametrization can be fine-tuned by the user for its specific dataset due to the fast UMAP layout computation of the structure components, although results overall are robust to small changes in these parameters. Parameters used for dbMAP embedding for individual studies and the integrated atlas, as well as those used for UMAP embedding of the atlas, are listed in Supplementary Table 3.

### Clustering

Clustering was performed by generating a k-nearest-neighbors graph from the structure components learned in dbMAP first step. For this, we applied the FindNeighbors function in Seurat with default parameters on the structure components. Clustering was then performed by a shared nearest neighbor (SNN) modularity-based clustering algorithm by using the FindClusters function in Seurat with default parameters.

### Identification of marker genes

Cluster marker genes were found with Seurat’s function FindAllMarkers, which finds differentially expressed genes between a cluster and all remaining cells with a Wilxocon rank sum test. Marker genes were then ranked by their log of fold change of expression in particular cell types. The two marker genes with the highest log of fold change for each cluster were chosen for the Dotplot and heatmap visualizations. A comprehensive table of all marker genes is provided as Supplementary Data.

### Cell-type annotation

Clusters were annotated to cell types corresponding the metadata of Madissoon et al.^28^ and the transferred labels from Travaglini et al. (https://doi.org/10.1101/742320) and accordingly to canonical marker gene expression.

### Doublet identification and removal

We identified five clusters that presented without specific marker genes and in spurious trajectories as doublets and removed them. Three clusters scattered the white space between alveolar cells, monocytes/macrophages, and lymphocytes in pairwise trajectories between these three cell types, effectively corresponding to doublets. The two remaining doublet clusters were scattered between mast cells and monocytes. We provide code for the identification and removal of these doublet clusters.

### Alveolar epithelia analysis and cell cycle scoring

For subsetting of the alveolar epithelia populations, we selected annotated alveolar cell clusters. The precomputed structure components were then used to perform another round of layout with dbMAP (parameters listed in Suppl. Table 1), followed by clustering as described for the complete atlas with parameters. A total of 22 subclusters were identified and annotated on basis of predicted cell types learnt from Travaglini et al. (https://doi.org/10.1101/742320) and marker gene expression as performed with the complete atlas. Seurat v3 built-in markers for the G1 and G2/M phases were performed to obtain a gene enrichment score that was used to automatically classify cells between S, G1 and G2/M states.

### Pseudotemporal ordering and differentiation dynamics

For lineage inference and pseudotime ordering of alveolar cells, we employed Palantir^36^. As for dbMAP, Palantir encodes diffusion structure, which is then exploited to compute pseudotime ordering, branching probabilities and differentiation potential of single cells with high-resolution, given a start cell. As discussed in our results, we selected either an MKI67+ AT2 cell belonging to the extremity of the Alveolar subtype 2.8 cluster, or an MKI67+ /KRT+ cell of the Basal cluster. Palantir was computed with identical parameters in both settings. Briefly, we computed Palantir’s multiscale space using 10 diffusion components and analyzing each cell 15 nearest neighbors, while using the default 500 waypoints for results estimation. Results were integrated into the Seurat workflow and visualized accordingly. As previously described, computation of gene expression trends was performed after imputation of the full expression matrix with MAGIC^62^ to smooth signal sparseness, and visualization was performed within Palantir.

### Generation of diagrams and illustrations

A licensed version of BioRender was employed to generate diagrams present in most figures.

### Visualization of gene expression

Visualization of gene expression was performed with dbMAP plots of gene expression and with dot plots. The Seurat functions FeaturePlot and DotPlot were applied to the integrated Seurat object with particular coloring thresholds for each gene so as to visualize expression throughout the color scale (Suppl. Table 4).

### Supplementary data

A Table with differentially expressed genes (DEG) for each cell type in the lung atlas is available as Lung_markers.csv. Differentially expressed genes (DEG) tables for each cell type in the lung atlas are available as Lung_markers.csv (Supplementary Data).

## Supplementary information

Supplementary Information 1.

## Data Availability

A downsized version of the integrated human atlas containing 10,000 randomly sampled cells is available as a Cerebro^63^ interactive webpage which can be easily explored by non-bioinformaticians at https://humanlung.iqm.unicamp.br . In Cerebro, users can readily visualize gene expression with dbMAP embeddings, search for each cluster differentially expressed genes, obtain functional enrichment scores for clusters of interest from a wide range of biomedical databases and export publication-level plots and tables. Fully processed data is also available as a complete Seurat object (.Rds) and as a Cerebro (.crb) file. All further data is available from the authors upon request.

## Code availability

All code used for analysis of single-cell RNA sequencing data is available at https://github.com/davisidarta/humanlung. dbMAP is available at https://github.com/davisidarta/dbMAP as a python library, which can also be easily used within R with the reticulate package. A docker image containing all necessary packages for analysis with an RStudio interface is also available.
