# Supplementary material for: SARS-CoV-2 receptor is co-expressed with elements of the kinin–kallikrein, renin–angiotensin and coagulation systems in alveolar cells
Source: Sci Rep. 2020 Nov 11;10:19522. doi: 10.1038/s41598-020-76488-2 (PMC7658217; doi:10.1038/s41598-020-76488-2)
Supplement: Supplementary file 1 — Supplementary Information 1. [file 41598_2020_76488_MOESM1_ESM.pdf]

**Supplementary data**

# SARS-CoV-2 receptor is co-expressed with elements of the kinin-kallikrein, renin-angiotensin and coagulation systems in alveolar cells

Davi Sidarta-Oliveira[1,2], Carlos Poblete Jara[1,3], Adriano J. Ferruzzi[4], Munir S. Skaf[4], William H. Velander[5], Eliana P. Araujo[1,3], Licio A. Velloso[1]

## Supplementary Figures

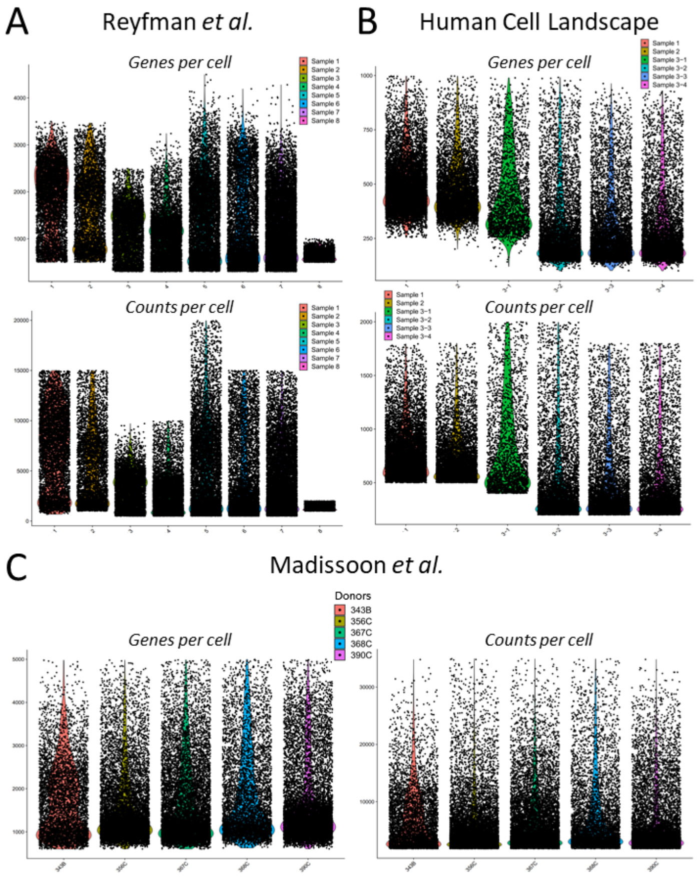


**Supplementary Figure 1. Quality control metrics for included data.** Violin plots of quality metrics (detected genes and RNA counts for each cell) showing each of the original datasets used for integration. **a.** Quality control metrics (QC) for Reyfman *et al*. data [1]. **b.** QC for the Human Cell Landscape lung data. **c.** QC for Madissoon *et al.* data [2].

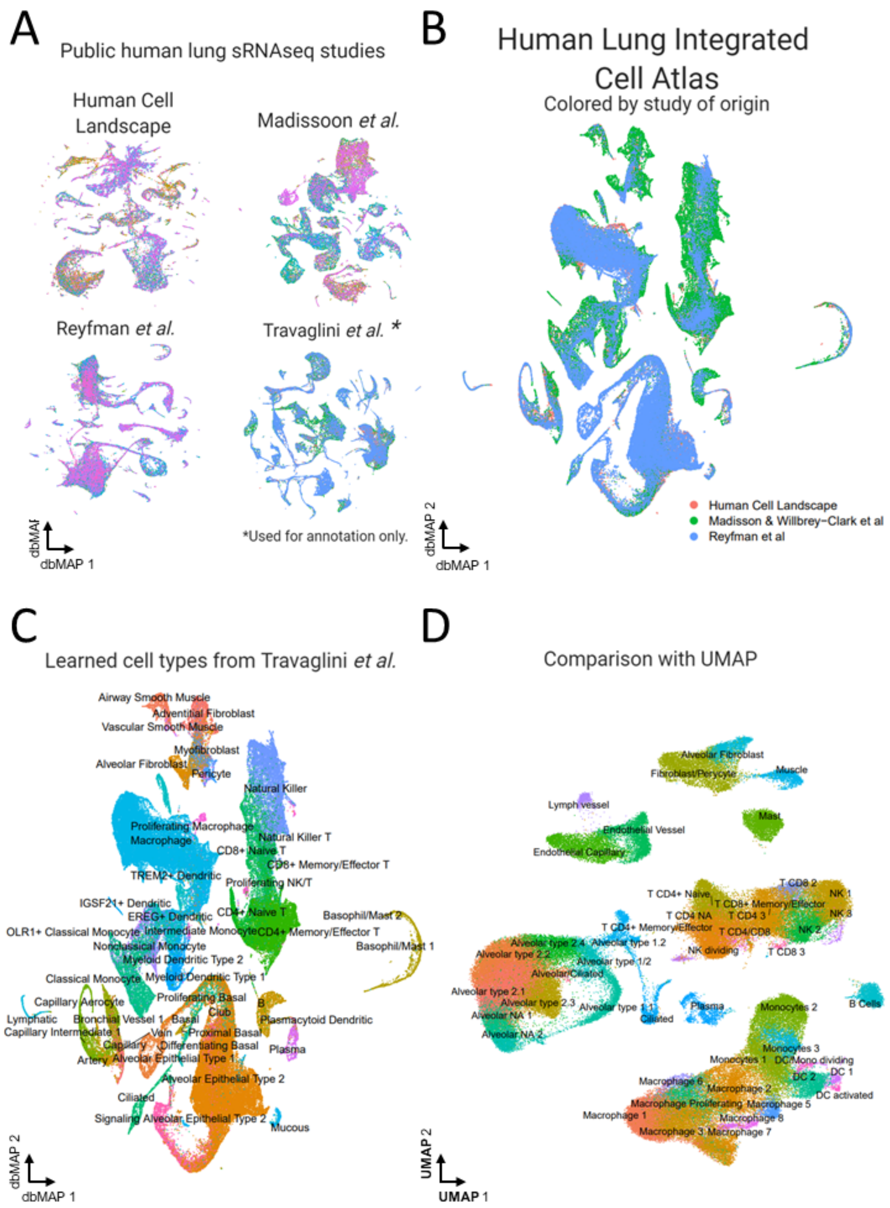


**Supplementary Figure 2. Details on integration, annotation and UMAP layout. a.** dbMAP embeddings of each individual batch-corrected study prior to integration and label transfer. **b.** dbMAP embedding of the Human Lung Integrated Cell Atlas. Cells were colored by their study of origin. Cell clusters were not particularly enriched for cells from a particular study, and overall integration is able to account for the weighted information from each study. **c.** Labels learned by transfer learning from Travaglini *et al*. annotations (https://doi.org/10.1101/742320). Annotation of resulting clusters and cell-type assignment was partly guided by these annotations. **d.** UMAP layout was computed on top 50 Principal Components after Principal Component Analysis (PCA), the default adopted workflow. Overall cluster configuration is similar between UMAP and dbMAP embeddings, being clearer that dbMAP is advantageous for the visualization of rare populations and differentiation trajectories, taking as example B cells, which are mapped in its differentiation trajectory into plasma cells, whereas UMAP embeds these clusters as completely apart populations. Clusters are annotated by cell type annotation.

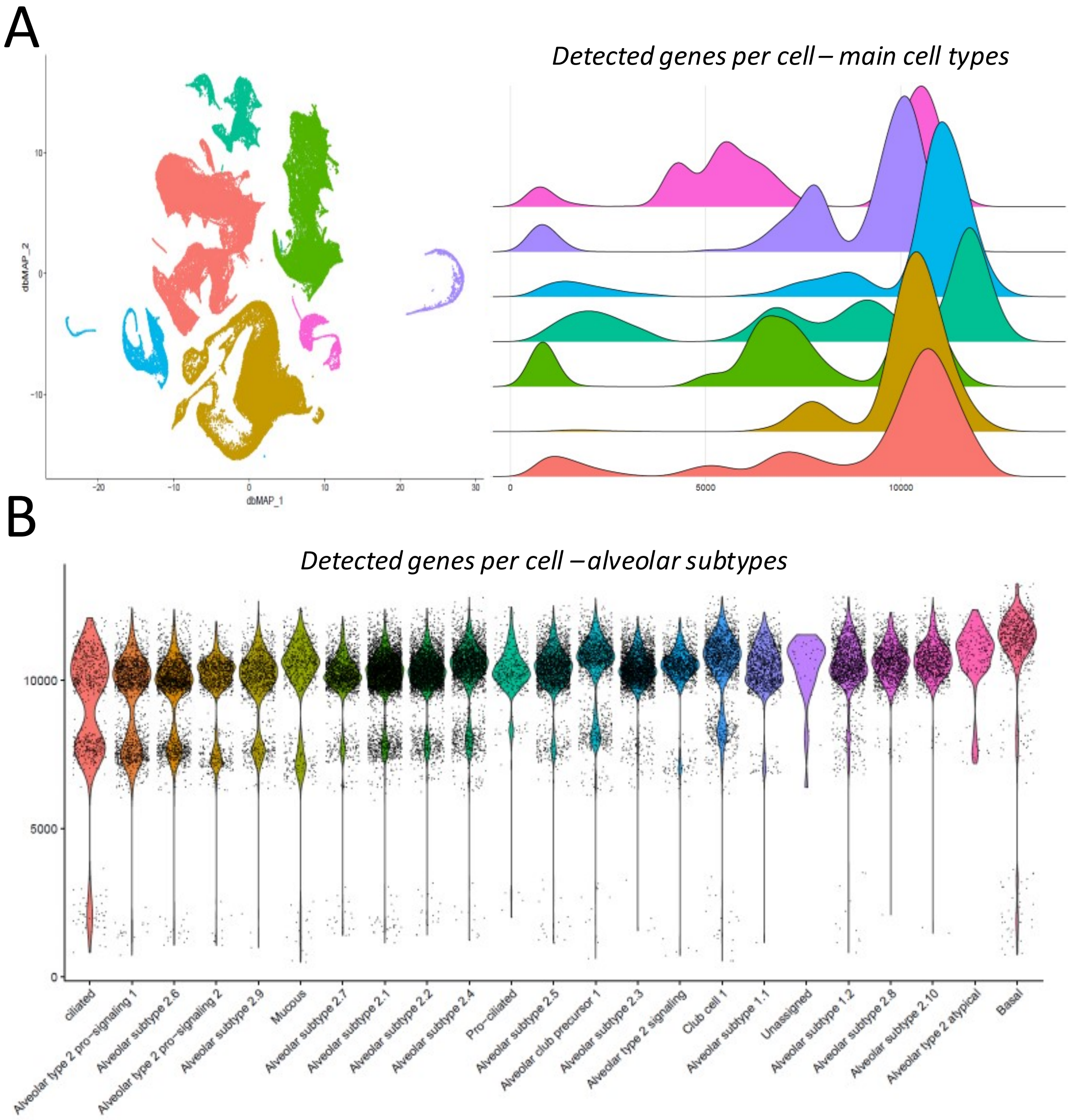


**Supplementary Figure 3. Visualization of library size for the corrected data clusters.** **a.** Left: Major cell types from the integrated atlas (alveolar, vessel, macrophages, fibroblasts/muscle, T cells, B and plasma cells, and mast cells) as visualized in the dbMAP embedding. Right: ridge plot showing the frequency distribution of detected genes per cell for each cell belonging to the major cell types identified. **b.** Violin plot representing the frequency distribution of detected genes per cell for each cell belonging to alveolar cell subclusters.

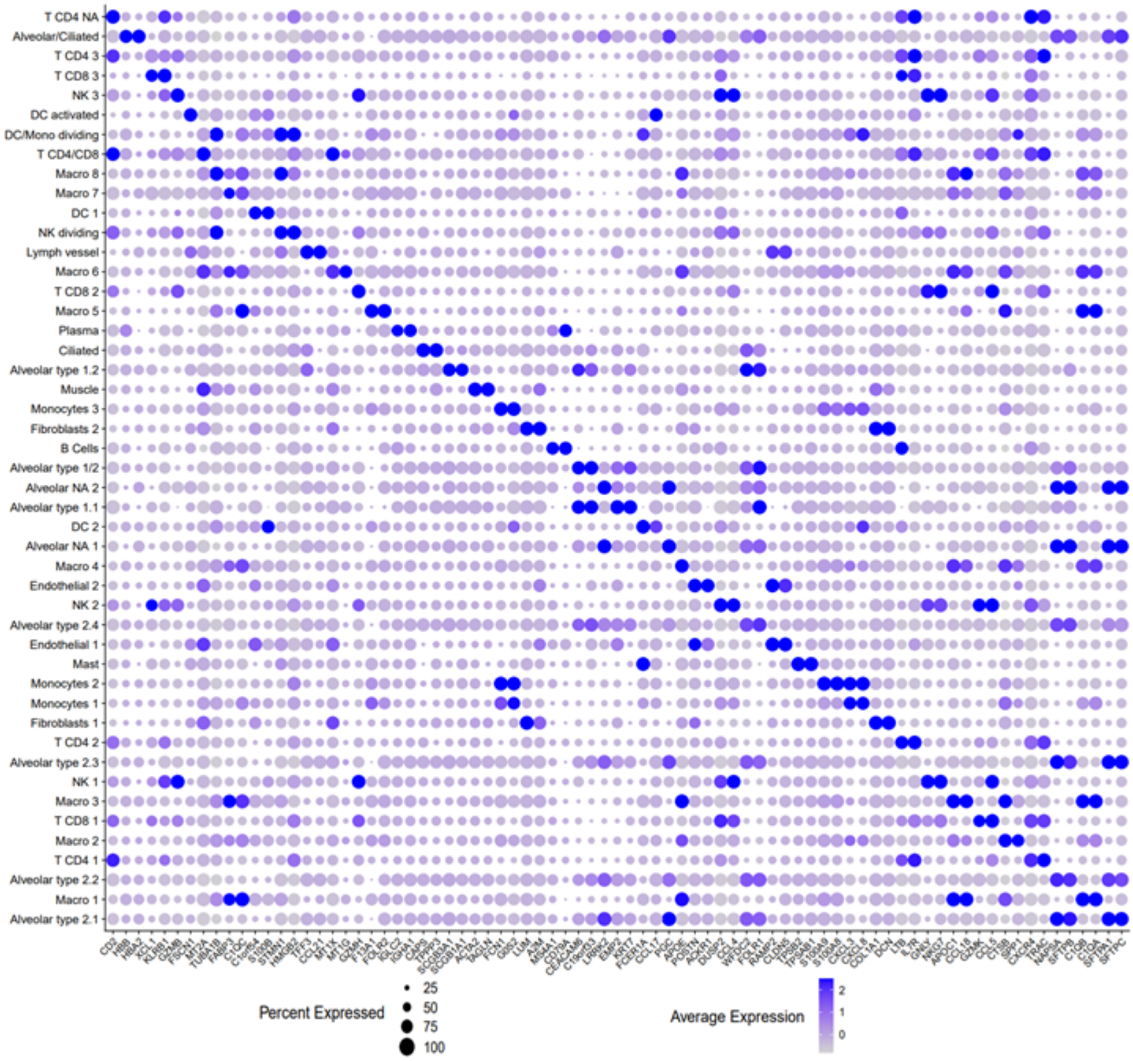

**Supplementary Figure 4. Dotplot of clusters gene expression markers.** Dot plot visualization of top 2 highest scoring markers per cell type. Larger circles mean a larger fraction of cells from a specific cell type express that gene, even though at exceptionally low rates. Darker circles mean the average gene expression for that gene in a specific cell-type is higher.

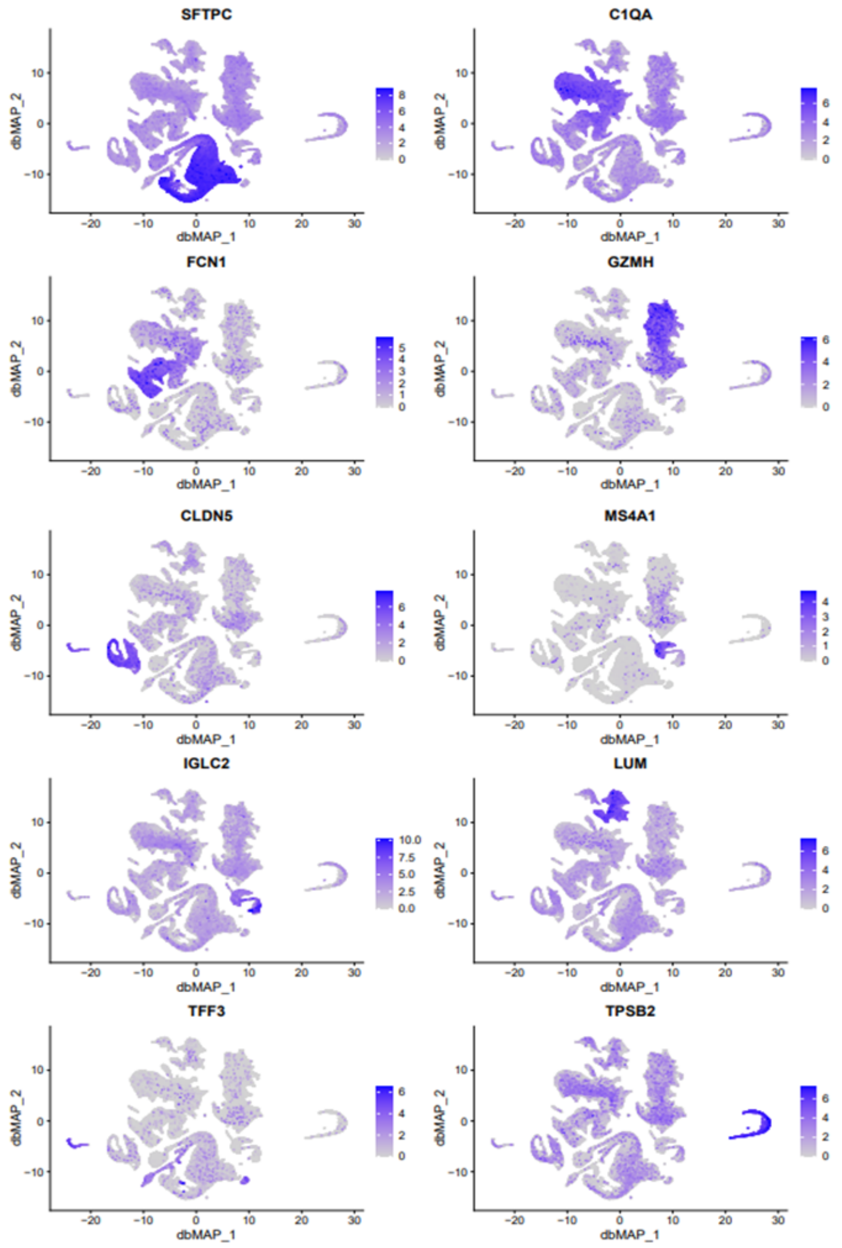


**Supplementary Figure 5. Panel of dbMAP embedded gene expression of 10 cell-type markers.** Visualization of gene expression in dbMAP embeddings of the lung atlas. SFTPC: alveolar cells. C1QA: macrophages. FCN1: monocytes. GZMH: T cells. CLDN5: endothelial and lymph vessels cells. MS4A1: B Cells. IGLC2: Plasma cells. LUM: Fibroblasts. TFF3: lymph vessel and alveolar ciliated cells. TPSB2: Mast cells. It is possible to generate similar plots for 20,000 genes on the atlas online database.

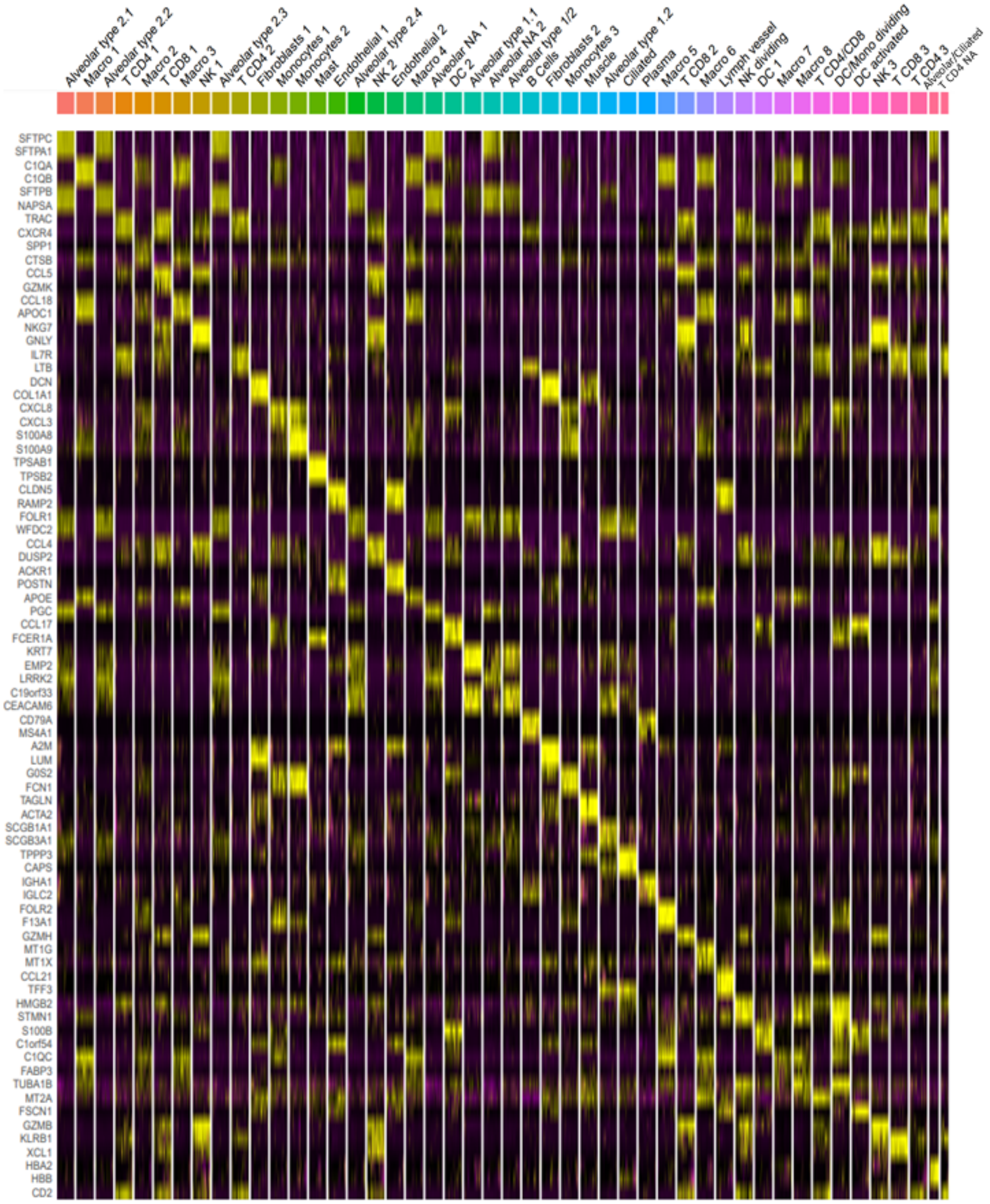


**Supplementary Figure 6. Heatmap of cell-type markers.** Heatmap of top two gene expression markers for each cell type cluster. For visualization, the cell number was downsampled by a factor of 100. Top annotations represent cell types.

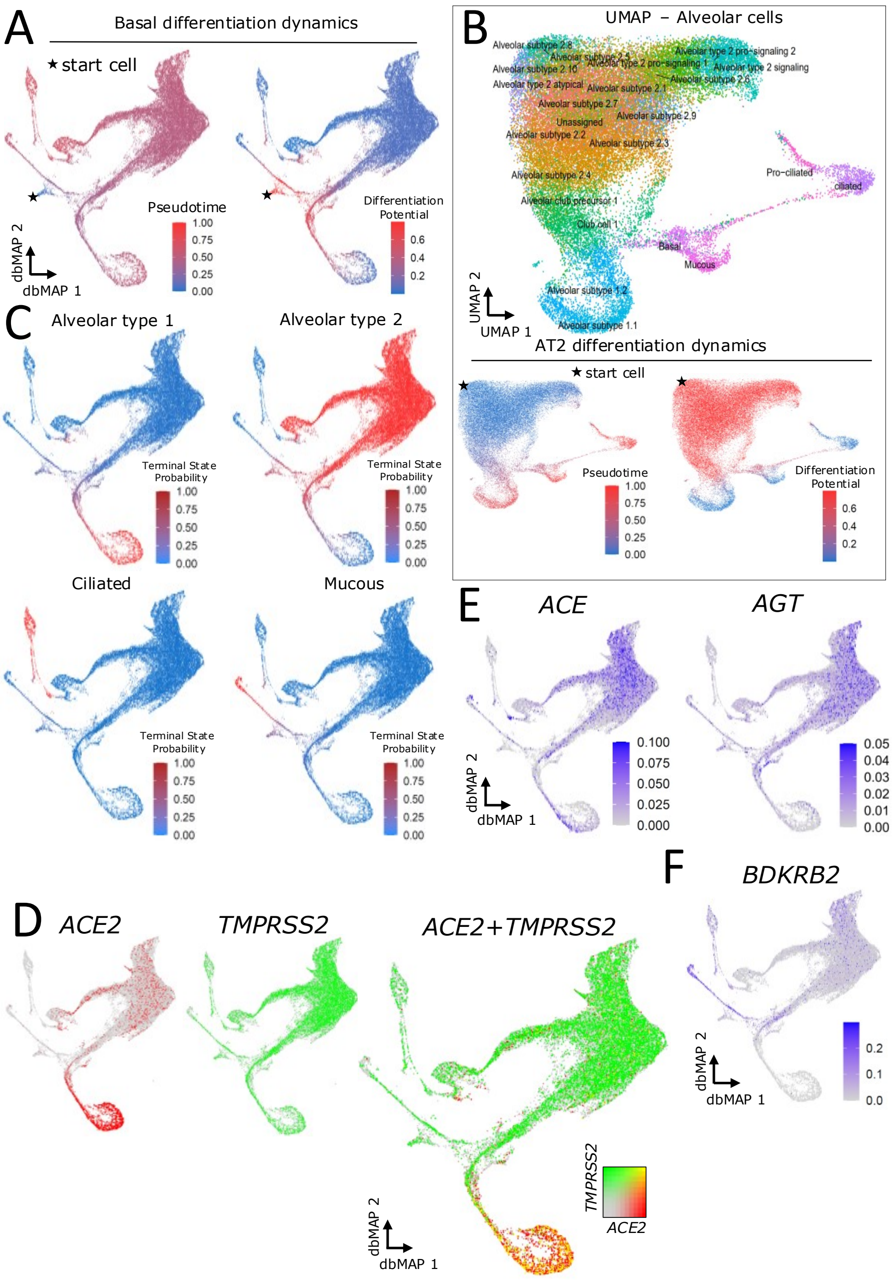


**Supplementary Figure 7. Basal cell progeny, UMAP comparison and additional gene expression visualization of the alveolar epithelia. a.** Basal cell differentiation dynamics. The star marks the start cell used in computations. Left: pseudotime ordering of single-cells. Note that for the basal cell progeny, pseudotime increases sharply as cells leave the start neighborhood. Right: differentiation potential of single-cells for the basal progeny. Note that cells belonging to the club-AT1 precursor cluster, some AT-signaling and pro-ciliated cells present with high differentiation potential, as in results from the AT2 progeny. **b.** UMAP embedding of alveolar cells. Cells are colored by their assigned cluster within the dbMAP analysis. Clusters are identified by overlaying annotations. **c.** Terminal state probabilities for the four identified terminal states: AT1, AT2, ciliated and mucous. **d.** Visualization of *ACE2* (red) and *TMPRSS2* (green) co-expression (yellow) in the dbMAP embedding of alveolar cells. As shown, AT1 cells most significantly co-express these genes, with fewer AT2 cells doing so. **e.** Visualization of *ACE* and *AGT* expression in the alveolar cells dbMAP embedding. **f.** Likewise, visualization of *BDKRB2* expression.

## Supplementary Tables

**Supplementary Table 1. Quality control inclusion criteria for cells from analyzed studies.** Cells were filtered by a minimum and maximum threshold of detected reads and detected genes so as to avoid overrepresentation of doublets, scRNAseq experimental artifacts that lead to the recognition of multiple cells as one (i.e., two cells in a single droplet). Cells with high mitochondrial gene expression are associated with low-quality reads and were removed from analysis. Each sample from Reyfman *et al.* [1] and Human Cell Landscape data was filtered separately, while Madissoon *et al.* [2] data was of extreme high-quality and presented very low batch-effects, therefore being filtered by a jointly defined threshold. Travaglini *et al.* (https://doi.org/10.1101/742320). data was used for annotation purposes only, and also filtered to a jointly defined threshold.

| | | | | |
|---|---|---|---|---|
| | **Reyfman *et al.*** | | | |
| Sample | **1** | **2** | **3** | **4** |
| Detected reads | > 500 & < 15,000 | > 1,000 & < 15,000 | > 500 & < 10,000 | > 500 & < 10,000 |
| Detected genes | >500 & < 3,500 | >500 & < 3,500 | > 300 & < 2,500 | > 300 & < 3,500 |
| % Mitochondrial Genes | < 20 | < 10 | < 10 | < 10 |
| | | | | |
| Sample | **5** | **6** | **7** | **8** |
| Detected reads | > 500 & < 20,000 | > 500 & < 15,000 | > 500 & < 15,000 | > 1,000 & < 2,000 |
| Detected genes | > 300 & < 4,500 | > 300 & < 5,000 | > 300 & < 4,500 | > 500 & < 1,000 |
| % Mitochondrial Genes | < 20 | < 10 | < 10 | < 11 |
| | | | | |
| | **Madissoon *et al.*** | | | |
| Detected reads | | >1,800 & < 35,000 | | |
| Detected genes | | > 600 & < 5,000 | | |
| % Mitochondrial Genes | | < 10 | | |
| | | | | |
| | **Human Cell Landscape** | | | |
| Sample | **1** | **2** | **3** | **4** |
| Detected reads | > 500 & < 1,800 | > 500 & < 1,800 | > 400 & < 2,000 | > 200 & < 2,000 |

| | | | | |
|---|---|---|---|---|
| Detected genes | > 250 & < 1,000 | > 200 & < 1,000 | > 100 & < 1,000 | > 100 & < 1,000 |
| % Mitochondrial Genes | < 20 | < 20 | < 20 | < 20 |
| | | | | |
| Sample | | **5** | **6** | |
| Detected reads | | > 200 & < 1,800 | > 200 & < 1,800 | |
| Detected genes | | > 100 & < 1,000 | > 100 & < 1,000 | |
| % Mitochondrial Genes | | < 20 | < 20 | |
| | | | | |
| | **Travaglini *et al.*** | | | |
| Detected reads | | > 1,000 & < 40,000 | | |
| Detected genes | | > 600 & < 5,000 | | |
| % Mitochondrial Genes | | < 10 | | |

**Supplementary Table 2.** Parameters used for dbMAP embedding for individual studies and the integrated atlas, as well as those used for UMAP embedding of the atlas. dbMAP takes four main parameters. During diffusion, a number **N** of structure components are computed accounting for each cell **K** nearest neighbors. After automatic scaling and selection of relevant components by eigengap analysis, a UMAP layout is generated with **M** as the effective minimum distance between embedded points and **S** as the effective scale of embedded points. Importantly, visualization parametrization can be fine-tuned by the user for its specific dataset due to the fast UMAP layout computation of the structure components, for example by changing the learning rate, although results overall are robust to small changes in these parameters.

| | **Integrated Atlas** | **Reyfman *et al.*** | **Madissoon *et al*.** | **Human Cell Landscape** |
|---|---|---|---|---|
| **UMAP** | | | | |
| n_PCs | 50 | N/A | N/A | N/A |
| min.dist | 0.5 | | | |
| spread | 1 | | | |
| | | | | |
| **dbMAP** | | | | |
| Computed DCs (N) | 300 | 300 | 200 | 300 |
| Selected DCs (automated) | 191 | 207 | 147 | 169 |
| *k*-nearest-neighbors (K) | 50 | 50 | 15 | 30 |
| min.dist (M) | 0.3 | 0.3 | 0.6 | 0.3 |
| spread (S) | 2 | 2 | 1 | 1.5 |
| Learning rate | 2 | 1.2 | 1 | 1 |

**Supplementary Table 3.** Coloring thresholds for plots obtained for each gene showed in the analysis. Plots were first visualized with a high threshold, which was decreased as little as possible, so as to visualize gene expression throughout the color scale. The combined thresholds used for the dotplots from figures 3, 4, 5 and 6 are also shown.

<table>
<tr><th>Plotting colorscale thresholds</th><th>ACE2</th><th>TMPRSS2</th><th>PIKFYVE</th><th>TPCN2</th><th>CTSL</th><th>KNG1</th><th>KLKB1</th><th>BDKRB2</th><th>ACE</th><th>BDKRB1</th></tr>
<tr><td>DimPlots</td><td>< 0.1</td><td>< 3</td><td>< 1</td><td>< 0.25</td><td>< 2</td><td>< 0.02</td><td>< 0.1</td><td>< 0.3</td><td>< 0.1</td><td>< 0.06</td></tr>
<tr><td>DotPlot Fig. 3</td><td colspan="5">None</td><td colspan="5"></td></tr>
<tr><td>DotPlot Fig. 4</td><td colspan="5"></td><td colspan="5">< 6</td></tr>
<tr><td></td><td>AGT</td><td>REN</td><td>AGTR1</td><td>SERPINE1</td><td>PLAT</td><td>FGG</td><td>SFTPC</td><td colspan="3" rowspan="4"></td></tr>
<tr><td>DimPlots</td><td>< 1</td><td>< 1</td><td>< 0.2</td><td>< 0.1</td><td>< 0.3</td><td>< 2</td><td>< 8</td></tr>
<tr><td>DotPlot Fig. 5</td><td colspan="3">< 3</td><td colspan="4"></td></tr>
<tr><td>DotPlot Fig. 6</td><td colspan="3"></td><td colspan="4">< 5</td></tr>
</table>

| Key Resources Table | | |
|---|---|---|
| **scRNAseq of human lung** | **Sequencing technology** | **Accession** |
| Reyfman *et al.* | 10X Genomics v2 | GEO GSE122960 |
| | | https://www.ncbi.nlm.nih.gov/geo/query/acc.cgi?acc=GSE122960 |
| Madissoon *et al.* | 10X Genomics v2 | NCBI BIOPROJECT PRJEB31843 |
| | | https://www.tissuestabilitycellatlas.org/ |
| Human Cell Landscape | Microwell-seq | GEO GSE134355 |
| | | https://www.ncbi.nlm.nih.gov/geo/query/acc.cgi?acc=GSE134355 |
| **Softwares and algorithms** | | |
| R 3.6.2 | R Core | https://www.r-project.org/ |
| Seurat 3.1.5 | Stuart and Butler *et al.* | https://satijalab.org/seurat/ |
| dbMAP v0.1 | Sidarta-Oliveira and Velloso | https://github.com/davisidarta/dbMAP |

**Key Resources Table.** Summary of all data and software used.

References

1. Reyfman, P.A.*, et al.* Single-Cell Transcriptomic Analysis of Human Lung Provides Insights into the Pathobiology of Pulmonary Fibrosis. *Am J Respir Crit Care Med* **199**, 1517-1536 (2019).
2. Madissoon, E.*, et al.* scRNA-seq assessment of the human lung, spleen, and esophagus tissue stability after cold preservation. *Genome Biol* **21**, 1 (2019).
